# Metabolic engineering and mechanical investigation of enhanced plant autoluminescence

**DOI:** 10.1111/pbi.14068

**Published:** 2023-05-08

**Authors:** Peng Zheng, Jieyu Ge, Jiayi Ji, Jingling Zhong, Hongyu Chen, Daren Luo, Wei Li, Bo Bi, Yongjun Ma, Wanghui Tong, Leiqin Han, Siqi Ma, Yuqi Zhang, Jianping Wu, Yanqiu Zhao, Ronghui Pan, Pengxiang Fan, Mengzhu Lu, Hao Du

**Affiliations:** ^1^ College of Agriculture and Biotechnology Zhejiang University Hangzhou China; ^2^ ZJU‐Hangzhou Global Scientific and Technological Innovation Center Zhejiang University Hangzhou China; ^3^ Hainan Institute of Zhejiang University Sanya China; ^4^ Marine Agriculture Research Center Tobacco Research Institute of Chinese Academy of Agricultural Sciences Qingdao China; ^5^ Key Laboratory of Structural Biology of Zhejiang Province, School of Life Sciences Westlake University, Westlake Laboratory of Life Sciences and Biomedicine, Institute of Biology, Westlake Institute for Advanced Study Hangzhou China; ^6^ State Key Laboratory of Subtropical Silviculture Zhejiang A&F University Hangzhou China

**Keywords:** Plant autoluminescence, FBP, energy, metabolic engineering, abiotic stress

## Abstract

The fungal bioluminescence pathway (FBP) was identified from glowing fungi, which releases self‐sustained visible green luminescence. However, weak bioluminescence limits the potential application of the bioluminescence system. Here, we screened and characterized a *C3′H1* (4‐coumaroyl shikimate/quinate 3′‐hydroxylase) gene from *Brassica napus*, which efficiently converts *p*‐coumaroyl shikimate to caffeic acid and hispidin. Simultaneous expression of *BnC3′H1* and *NPGA* (null‐pigment mutant in *A. nidulans*) produces more caffeic acid and hispidin as the natural precursor of luciferin and significantly intensifies the original fungal bioluminescence pathway (oFBP). Thus, we successfully created enhanced *FBP* (*eFBP*) plants emitting 3 × 10^11^ photons/min/cm^2^, sufficient to illuminate its surroundings and visualize words clearly in the dark. The glowing plants provide sustainable and bio‐renewable illumination for the naked eyes, and manifest distinct responses to diverse environmental conditions via caffeic acid biosynthesis pathway. Importantly, we revealed that the biosynthesis of caffeic acid and hispidin in *eFBP* plants derived from the sugar pathway, and the inhibitors of the energy production system significantly reduced the luminescence signal rapidly from *eFBP* plants, suggesting that the FBP system coupled with the luciferin metabolic flux functions in an energy‐driven way. These findings lay the groundwork for genetically creating stronger *eFBP* plants and developing more powerful biological tools with the FBP system.

## Introduction

Bioluminescence from living organisms emitting light visible to naked human eyes is a natural phenomenon, which has been observed in bacteria, jellyfish, earthworms, fireflies and fungi (Fleiss and Sarkisyan, 2019; Love and Prescher, 2020; Oliveira *et al*., 2015; Tsarkova *et al*., 2016; Yeh and Ai, 2019). Artificial integration of natural bioluminescent reactions into living systems has also become a reporting tool widely used in molecular and cell biology (Yeh and Ai, 2019). However, some natural bioluminescent systems still have significant disadvantages in their invisibility to the naked eye and reliance on advanced instruments, which limit their more widespread applications.

Although luciferin and luciferase could be delivered into plants via nanoparticles to emit bright bioluminescence, this approach is currently expensive and not self‐sustainable (Kwak *et al*., 2017). Recently, Kotlobay *et al*. (2018) have identified an FBP consisting of four genes: *NnHispS* (hispidin synthase), *NnH3H* (hispidin‐3‐hydroxylase), *NnLuz* (luciferase) and *NnCPH* (caffeylpyruvate hydrolase) from the bioluminescent mushroom *Neonothopanus nambi*. The FBP functions by converting caffeic acid to hispidin catalysed by HispS, followed by the conversion of hispidin to luciferin 3‐hydroxyhispidin through the activity of H3H. Then Luz converts luciferin to an unstable high‐energy intermediate (Kaskova *et al*., 2017), which emits light in the green spectrum (*λ*_max_ ~520 nm) and produces caffeylpyruvic acid. Then the caffeylpyruvic acid is converted to caffeic acid by CPH, recycling the caffeic acid for self‐sustained luminescence, which significantly extended the light emission continuously. *Rhodobacter capsulatus* tyrosine ammonia lyase (RcTAL) and two *Escherichia coli* 4‐hydroxyphenylacetate 3‐monooxygenase components (HpaB, HpaC) together could catalyse caffeic acid synthesis from tyrosine (Khakhar *et al*., 2020; Kotlobay *et al*., 2018; Mitiouchkina *et al*., 2020; Zhou *et al*., 2021). *RcTAL, HpaB* and *HpaC* genes have been introduced into FBP‐expressing mammalian cells (Mitiouchkina *et al*., 2020) or transiently infiltrated into leaves of an *N. benthamiana* line stably expressing the FBP with a moderate increase of luminescence (Khakhar *et al*., 2020). Notably, caffeic acid is a common intermediate in the phenylpropanoid pathway across plant species (Maeda and Dudareva, 2012; Vogt, 2010). In transgenic *N. tabacum FBP* plants, the level of caffeic acid is slightly lower than that in wild‐type plants (Mitiouchkina *et al*., 2020). It is possible that genetic engineering of plant genes in the phenylpropanoid biosynthesis pathway could generate brighter glowing *FBP* plants without the exogenous addition of caffeic acid (Barros *et al*., 2019; Khakhar *et al*., 2020; Maeda and Dudareva, 2012; Mitiouchkina *et al*., 2020; Vogt, 2010), making the fungal bioluminescent system attractive in numerous applications of bioimaging in detecting hormonal, stress, pathogenic, developmental and chemical stimuli and bio‐sustainable glowing ornamental plants.

Here, we successfully created enhanced autoluminescent plants by integrating C3′H, NPGA with the original *FBP* (*oFBP*) module. Moreover, we first characterized the biosynthesis of caffeic acid and hispidin, the precursor of luciferin, is energy dependent. Our finding provides novel strategies to optimize the bioluminescent systems in plants and enable further powerful applications.

## Results

### Characterization of C3′H and NPGA in boosting caffeic acid and hispidin biosynthesis

**Figure 1:**
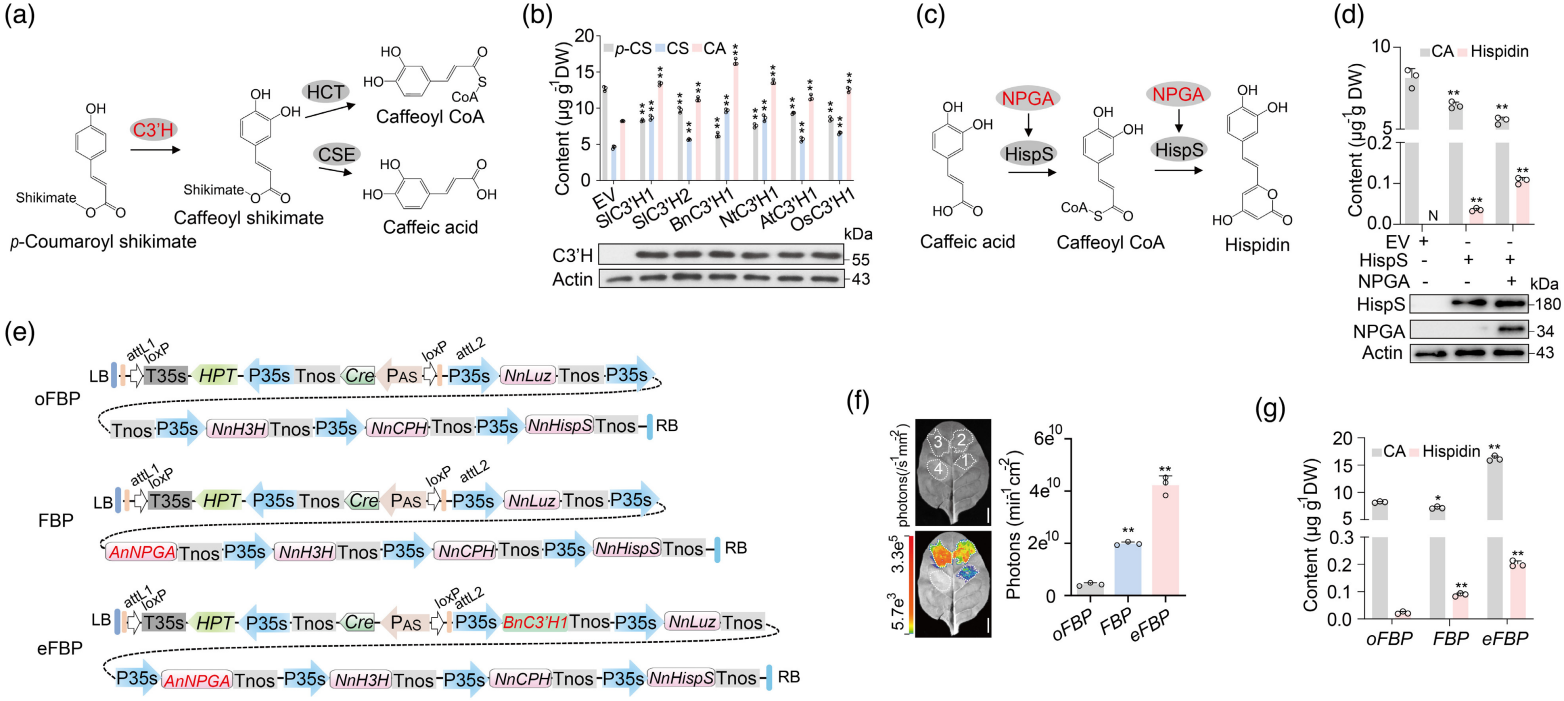
Mechanistic basis of C3′H and NPGA in enhancing luminescence emission. (a) Suggested mechanisms for C3′H involved in caffeoyl CoA and caffeic acid biosynthesis. C3′H, 4‐coumaroyl shikimate/quinate 3′‐hydroxylase; HCT, 4‐hydroxycinnamoyl CoA:shikimate/quinate hydroxycinnamoyltransferase; CSE caffeoyl shikimate esterase. (b) Mass spectra of caffeic acid produced by an *in vitro* enzyme assay using C3′H from different species. Three compounds of *p*‐Coumaroyl shikimate (*p*‐CS), caffeoyl shikimate (CS) and caffeic acid (CA) extracted from tobacco leaves transiently expressing indicated compounds were detected by standards, and empty vector (EV) was used as a control. C3′H‐Flag transient expression in tobacco leaves was determined by immunoblot analyses. (c) Proposed reaction catalysed by HispS and NPGA. HispS, hispidin synthase; NPGA, 4′‐phosphopantetheinyl transferase. (d) LC–MS/MS analysis of the content of Hispidin and Caffeic acid (CA) from injected tobacco leaves. HispS and NPGA transiently expressed in tobacco leaves were determined by immunoblot analyses. (e) The structure of binary constructs containing selectable marker HPT/marker‐excision Cre/loxP cassette (driven by P_AS_, anther‐specific promoter in tobacco). oFBP (1, original FBP), FBP (2) and eFBP (3, enhanced FBP) constructs are represented original bioluminescence, bioluminescence and enhanced bioluminescence, respectively. (f) Infiltration of WT tobacco leaves with oFBP, FBP and eFBP modules respectively. Bioluminescent intensity analysis from infiltrated leaves after 48 h. *EV* (4), empty vector. (g) Analysis of the content of hispidin and caffeic acid (CA) from infiltrated leaves after 48 h. Scale bars, 1 cm. Error bars indicate means ± SD (*n* = 3). Statistical significance was assessed using two‐tailed *t*‐tests (*P ≤ 0.05, **P ≤ 0.01).

To increase caffeic acid in FBP system, we focused on characterizing the caffeic acid and hispidin biosynthesis process in plants, as an insufficient supply of caffeic acid could limit hispidin biosynthesis, which is the bottleneck of the FBP cycle (Barros *et al*., 2019; Maeda and Dudareva, 2012; Mitiouchkina *et al*., 2020; Vogt, 2010). Caffeic acid and caffeoyl CoA are cinnamic acid derivatives naturally abundant in all plant species and is primarily involved in the biosynthesis of lignin and flavonoids (Barros *et al*., 2019; Maeda and Dudareva, 2012; Vogt, 2010). In the pioneering research, HispS enzyme was introduced to produce hispidin using caffeic acid and caffeoyl CoA as direct substrates (Mitiouchkina *et al*., 2020). The membrane‐associated C3′H is the key enzyme in the ferulate pathway, and catalyses 3‐hydroxylating p‐coumaroyl derivatives to the corresponding caffeic acid conjugates including caffeic acid and caffeoyl CoA (Gou *et al*., 2018; Schoch *et al*., 2001; Zhang *et al*., 2022) (Figure 1a). Thus, we supposed that C3′H facilitates the biosynthesis of caffeic acid and hispidin.

We further identified putative C3′H encoding gene orthologues via phylogenetic analyses in a wide range of plant species (Figure S1). Enzyme activities of 14 C3′H1s from different plant species were assessed by comparing the binding free energies of C3′H1/*p*‐coumaroyl shikimate complex. The optimal results of each molecular docking showed that BnC3′H1 from *Brassica napus* has generally lower binding energy with *p*‐coumaroyl shikimate than other C3′H1s (Table S1), suggesting that BnC3′H1 may have relatively higher activity of 4‐coumaroyl shikimate/quinate 3′‐hydroxylase.

Then, we made a set of expression constructs to evaluate the enzyme activity of C3′Hs from different plant species *in vivo*. Using *Agrobacterium*‐mediated transiently expressed C3′Hs in the leaves of *N. tabacum* (Figure S2a,c), we found that overexpressing BnC3′H1 led to a bit higher catalytic activity than other C3′Hs, suggesting that BnC3′H1 produces caffeoyl shikimate and caffeic acid more efficiently (Figure 1b). To probe the mechanistic basis of C3′H in converting *p*‐coumaroyl shikimate to caffeic acid and caffeoyl CoA (unstable intermediate), we modelled the structure of BnC3′H1 and tried molecular docking with an optimized process based on a published method (Jumper *et al*., 2021). The top‐ranking docking model of the BnC3′H1 and *p*‐coumaroyl shikimate suggested that four residues Trp112, His242, Trp294 and Thr298 in BnC3′H1 formed hydrogen bonds with *p*‐coumaroyl shikimate (Figure S3). Multiple protein sequence alignments showed that the four residues in C3′Hs involved in the catalysis are conserved across plant species (Figure S4), which suggested that the hydroxylation mechanism of C3′H linking the biosynthesis of caffeic acid conjugates is conserved.

To further boost the synthesis of hispidin in the bioluminescent plants, we attempted to enhance the catalytic ability of HispS. NPGA, a null‐pigment mutant from *A. nidulans*, was identified as the 4′‐phosphopantetheinyl transferase (PPTase) enzyme, which posttranslationally modifies modular and iterative synthase in a processive fashion, as well as enhances the activity of polyketide synthases (PKS) (Keszenman‐Pereyra *et al*., 2003; Marquez‐Fernandez *et al*., 2007; Mootz *et al*., 2002). HispS, the polyketide synthase family, produce secondary metabolites in a variety of organisms (Robbins *et al*., 2016) and is supposed to promote hispidin biosynthesis (Figure 1c). In the two previous studies, researchers integrated *NPGA* together with *FBP* genes into the genome of *P. pastoris* and tobaccos to emit observed light (Khakhar *et al*., 2020; Kotlobay *et al*., 2018). Although they used *NPGA* to create bioluminescent yeast and tobacco, there is still a lack of direct experimental evidence to dissect whether *NPGA* gene from *A. nidulans* could advance the synthesis of luminescent precursors hispidin in plants. To probe this puzzle, we used *Agrobacterium*‐mediated transiently expressed *NPGA* and *HispS* in leaves of *N. tabacum* (Figure S2b,c), which were subsequently used for LC–MS/MS analysis. Our data showed that even though HispS alone can convert caffeic acid to hispidin, it catalyses caffeic acid to synthesize hispidin 3 times more efficiently when used together with NPGA (Figure 1d). These data demonstrated that *BnC3′H1* and *NPGA* efficiently boosted caffeic acid and hispidin biosynthesis in enhancing plant bioluminescence.

### Creating enhanced *FBP* plants

The possibility that the exogenous addition of caffeic acid and hispidin could enhance plant luminescence in tobacco (Mitiouchkina *et al*., 2020) promoted us to design an enhanced fungal bioluminescence pathway (eFBP) via increasing the biosynthesis of caffeic acid and hispidin in plants. A previously described multiple genes assembly system TransGene stacking II with Cre recombinase/loxP‐mediated recombination (Zhu *et al*., 2017) was used to generate *oFBP*, *FBP* and *eFBP* DNA modules respectively (Figure 1e). Here, we identified an anther‐specific promoter (PAS) to yield marker‐free cassette by deleting plant‐selectable marker HPT (hygromycin phosphotransferase) and Cre, flanked by two loxP sites and two sites (attB1, attB2) for Gateway recombination (Figures S5a,b and S8a). The previously reported *oFBP* DNA module was used as a control. 48 h after being injected with *FBP* DNA modules in *Nicotiana tabacum* leaves for transient expression, *eFBP* DNA modules displayed the highest bioluminescent signals, roughly 8 times higher than those in *oFBP* modules (Figure 1e and Figure S5c). In addition, we measured the level of caffeic acid and hispidin in transiently expressed tobacco leaves infiltrated with *oFBP*, *FBP* and *eFBP* modules, respectively. The LC–MS/MS data illustrated that *FBP* produced about 3 times the amount of hispidin compared to *oFBP* (Figure 1f), and *eFBP* accumulated more than 2.2 times hispidin compared to *FBP* leaves. The result further provides physiological evidence of the important roles of BnC3′H1 and NPGA in enhancing plant bioluminescence.

**Figure 2:**
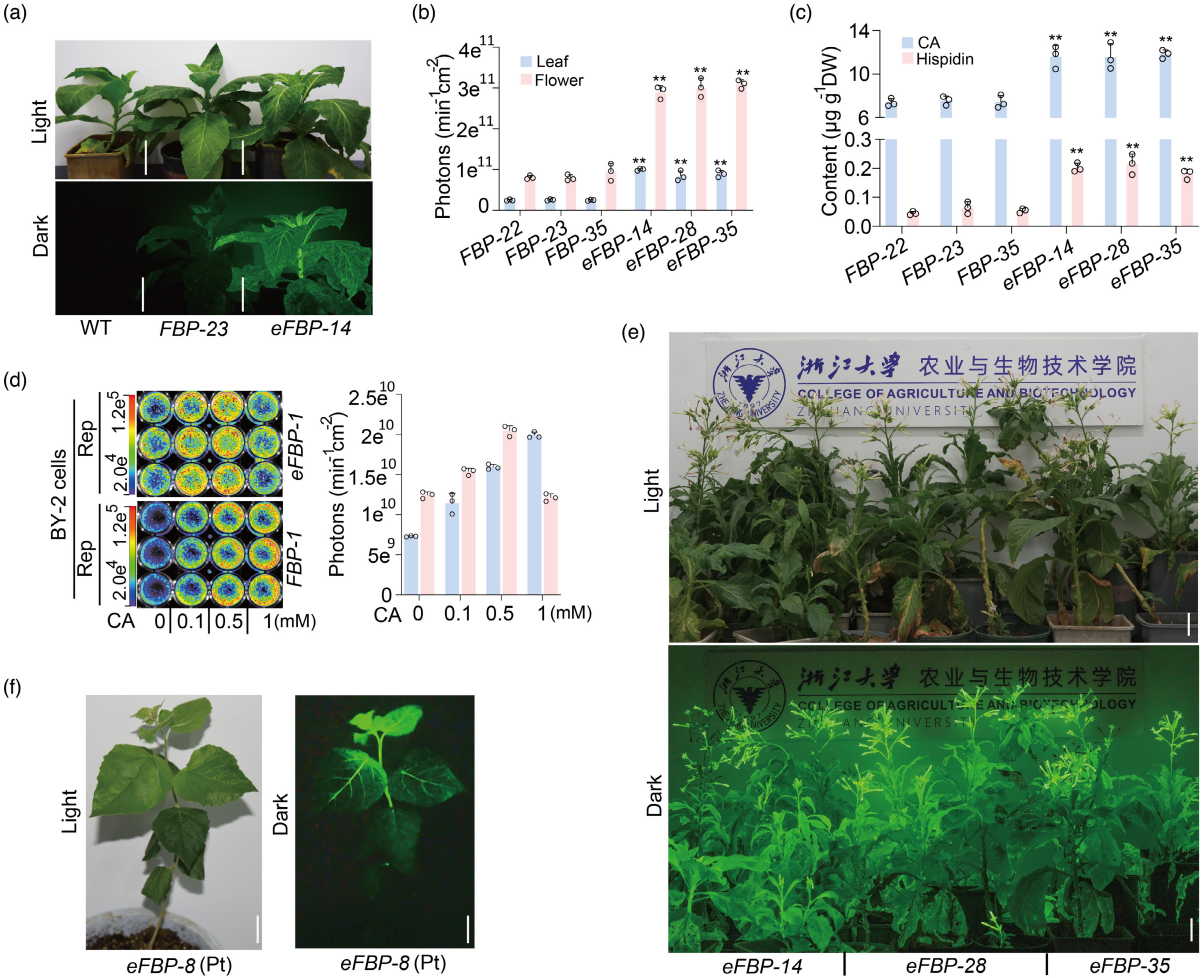
Creation of bioluminescent plants. (a) Appearance of *FBP* and *eFBP* transgenic lines at 60 DAG (day after germination) under ambient light with 1/200 s exposure, with 60‐s exposure under the dark, respectively. Glowing plants were captured with a Nikon D750 camera with AF‐S17‐35 mm F2.8D ED‐IF at ISO 2000, F6.3, 60‐s shutter speed. Wild type (WT) was used as control. Scale bars, 10 cm. (b) Statistical analysis of average photons emission from leaves and flowers of three independent *FBP* and *eFBP* lines. (c) LC–MS/MS analysis of caffeic acid and hispidin contents in leaves from *FBP* and *eFBP* transgenic seedlings. Error bars indicate means ± SD (*n* = 3). Statistical significance was assessed using two‐tailed *t*‐tests (*P ≤ 0.05, **P ≤ 0.01). (d) Representative bioluminescent image of *FBP* and *eFBP* transgenic BY‐2 cells fed with solution of gradient caffeic acid level. And measurement of average photons emission from exogenous caffeic acid fed *FBP* and *eFBP* transgenic BY‐2 cells by photographic instrument. (e) The performance of *eFBP* bioluminescent plants in lighting up the room, picture captured with a Nikon D750 camera with the above parameter. Scale bars, 10 cm. (f) Appearance of *eFBP* transgenic poplar sapling captured with a Nikon D750 camera with AF‐S17‐35 mm F2.8D ED‐IF at ISO 2000, F6.3, 5‐min shutter speed. Scale bars, 1 cm.

Thus, *FBP* and eFBP were subject to further characterization. Using *Agrobacterium*‐mediated transformation, we created *FBP* and *eFBP* transgenic tobacco lines. After screening by photographic instrument (Figure S6a) and real‐time‐quantitative PCR (RT‐qPCR) assay (Figure S6b), we successfully obtained the comparable expression level of five FBP genes (*AnNPGA*, *NnHispS*, *NnH3H*, *NnLuz* and *NnCPH*) with observed autoluminescence from *FBP* and *eFBP* transgenic tobacco lines (Figure S6a,b). The level of brightness allowed us to capture light‐emitting plant images in the dark by consumer‐grade cameras (Figure 2a and Figure S7a). Impressively, the autoluminescence from the glowing plants was visualized by a commercial video camera immediately in the dark room (Video S1). The maximal intensity reaching 3 × 10^11^ photons/min/cm^2^ from the *eFBP* flowers in three independent lines, the intensity of average photons roughly 3 times higher than those from *FBP* plants (Figure 2b and Figure S7b). Interestingly, the enhanced glowing plants did not display developmental differences in height and flowering time (Figure S7c,d), indicating that the integrated extrinsic metabolic pathway has limited effect on plant developmental process. Future studies will determine whether our fortified *eFBP* DNA module can also enable higher intensity of bioluminescence in other plant species.

We carried out LC–MS/MS analysis to evaluate whether the enhanced brightness from *eFBP* lines was due to increased luminescent metabolites, caffeic acid and hispidin, generated by the heterologously expressed *BnC3′H1* gene. It was evident that caffeic acid and hispidin levels in *eFBP* seedlings increased 1.6 times and 2.9 times respectively, compared with those in *FBP* seedlings (Figure 2c). The findings indicated that sufficient caffeic acid and hispidin accumulation boosted the FBP cycle and led to more photons emitted from *eFBP* plants. Further PCR analysis with T3 generation seedlings showed that the selectable marker/Cre gene cassette was excised as expected, producing marker‐free lines (Figure S8b,c), which was useful for next‐round metabolic engineering to create stronger autoluminescent plants. To confirm that enhanced caffeic acid availability is related to stronger light emission, glowing *N. tabacum* BY‐2 (bright‐yellow‐2) cell culture with the *FBP* or *eFBP* DNA module were developed. We observed approaching 3‐fold increase in light emission from three independent *eFBP* cell lines, which was strikingly higher than those from *FBP* cell lines (Figure S9a,b). By adding exogenous caffeic acid with gradually increasing concentrations to feed stable *FBP* and *eFBP* BY‐2 cell lines, we found that the *FBP* BY‐2 cells exhibited the strongest light emission at 1 mm caffeic acid addition, whereas the *eFBP* BY‐2 cells reached the highest level at 0.5 mm addition (Figure 2d). These results indicated that enhancing caffeic acid metabolic flux resulted in increased luminescence in *eFBP* transgenic lines, whereas, excessive accumulation of caffeic acid probably caused low toxicity to BY‐2 cells.

Previous studies reported that FBP worked via tissue transient expression in tomato, *Arabidopsis*, dahlia, *Catharanthus roseus*, and rose besides tobacco plants, as caffeic acid is ubiquitously present in higher plants (Barros *et al*., 2019; Khakhar *et al*., 2020; Maeda and Dudareva, 2012; Mitiouchkina *et al*., 2020; Vogt, 2010). To test whether the *eFBP* DNA module is applicable in other plant species for wider applications, we transiently expressed the *eFBP* DNA module in multiple plant species through *Agrobacterium* infiltration. We observed robust autoluminescence signals in the leaves and petals of *N. benthamiana* and two ornamental plants, *Phalaenopsis aphrodite* and *Chrysanthemum morifolium* (Figure S10a–c). Intriguingly, the flowering of *eFBP* tobacco plants emitted bright light that was sufficient to illuminate its surroundings and visualize words clearly in the dark (Figure 2e). The finding inspired us to explore whether the *eFBP* DNA module was functional in macrophanerophytes commonly planted along city streets. We engineered transgenic poplar, which is widely used as roadside trees in China, with the *eFBP* DNA module. After phenotypic and molecular screens, we successfully created autoluminescent poplar saplings (Figure 2f and Figure S11a,b). These data further support the universal application of the FBP system in herbaceous species and perennial woody plants.

### Abiotic stresses regulate the biosynthesis of caffeic acid in *eFBP* plants

**Figure 3:**
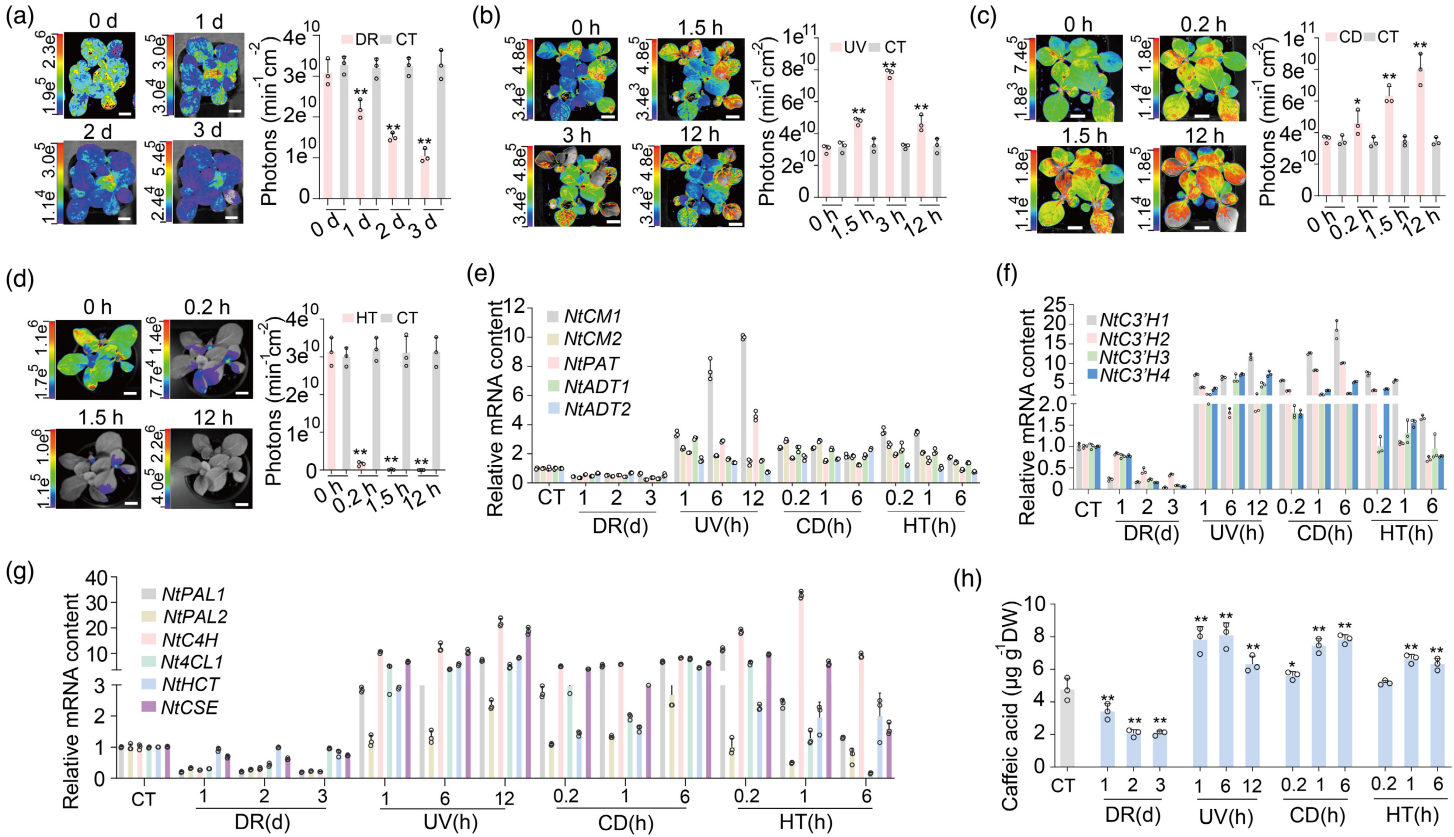
Perturbation of the eFBP system in tobacco. (a–d) The bioluminescent images and statistical analyses of photons emission from *eFBP* transgenic seedlings under various abiotic stresses containing drought, DR (a), ultraviolet, UV (b), cold 4 °C, CD (c), high temperature 37 °C, HT (d). (e–g) The expression profiles of *NtCMs*, *NtPAT*, *NtADTs* (e), *NtC3′H1‐4* (f), and *NtPALs*, *NtC4H*, *Nt4CL*, *NtHCT*, *NtCSE* (g) by RT‐qPCR, samples from wild‐type *N. tabacum* with different treatments including control (CT), drought (DR), ultraviolet (UV), cold 4 °C (CD), high temperature 37 °C (HT) at multiple time courses. d, day. h, hour. (h) Total caffeic acid amount in wild‐type *N. tabacum* seedlings after treatments as above. Scale bars, 1 cm. Error bars indicate means ± SD (*n* = 3). Statistical significance was assessed using two‐tailed *t*‐tests (*P ≤ 0.05, **P ≤ 0.01).

Because *eFBP*‐based plant autoluminescence has a wide range of potential applications from basic biology and biosensors to lighting and decoration of cities, houses, roads and landscape facilities, we investigated the influence of the plant surrounding environment on the *eFBP* system in plants. To test the intensity of *eFBP*‐based luminescent plants in the ever‐changing natural environment, we treated *eFBP* lines with different abiotic stresses which frequently occur in the natural environment. Our data clearly showed that mild to severe drought stresses significantly repressed the luminescence signal (Figure 3a and Figure S12a). By contrast, the luminescence signal of stressed leaves increased markedly after UV irradiation and cold stress (Figure 3b,c and Figure S12b,c). A high temperature at 37 °C led to a cliff‐like drop of luminescence signal after 0.2 h treatment (Figure 3d and Figure S12d). Although previous data reported that stresses, including injury and methyl jasmonate or ethylene, promoted the luminescence signal in *FBP*‐based transgenic tobacco, our observations based on three independent transgenic *eBFP* lines revealed that different stresses had distinct effects. We inferred that abiotic stresses exhibited impact via the caffeic acid biosynthetic pathway. Our RT‐qPCR assay and metabolite analysis illustrated that drought stress generally repressed transcripts of caffeic acid biosynthesis genes *NtCMs* (chorismate mutase), *NtPAT* (prephenate‐aminotransferase), *NtADTs* (arogenate dehydratase), *NtC3′Hs* (4‐coumaroyl shikimate/quinate 3′‐hydroxylase), *NtPALs* (phenylalanine ammonia‐lyase), *NtC4H* (cinnamic acid 4‐hydroxylase), *Nt4CLs* (4‐hydroxycinnamate:CoA ligase), *NtHCT* (4‐hydroxycinnamoyl CoA:shikimate/quinate hydroxycinnamoyltransferase) and *NtCSE* (caffeoyl shikimate esterase), consequently leading to a declined accumulation of caffeic acid in *N. tabacum* (Figure 2e–h). In contrast, transgenic seedlings treated with UV irradiation and cold displayed increased light emission, which correlated with higher expression of caffeic acid biosynthesis genes and more caffeic acid accumulation (Figure 2e–h). However, even though high temperature induced the accumulation of caffeic acid biosynthesis genes quickly at 0.2 h and 1 h, and the caffeic acid level gradually accumulated at 1 h and 6 h, the light signal was rapidly decreased under high temperature (Figure 2d–h and Figure S12d). The paradox could probably be explained by the rapid decline of luciferase enzymatic activity at higher temperature, which is consistent with the finding that luciferase loses activity at temperatures above 30 °C (Kotlobay *et al*., 2018). To avoid the circadian effects on bioluminescence observed in stress‐treated *eFBP* plants, parallel control under normal conditions displayed no marked change at the detected time course (Figure S12e,f). These features would enable us to utilize and continuously optimize the novel eFBP system in developing glowing plant varieties and powerful molecular tools.

### The metabolically coupled FBP cycle in plants are energy dependent

**Figure 4:**
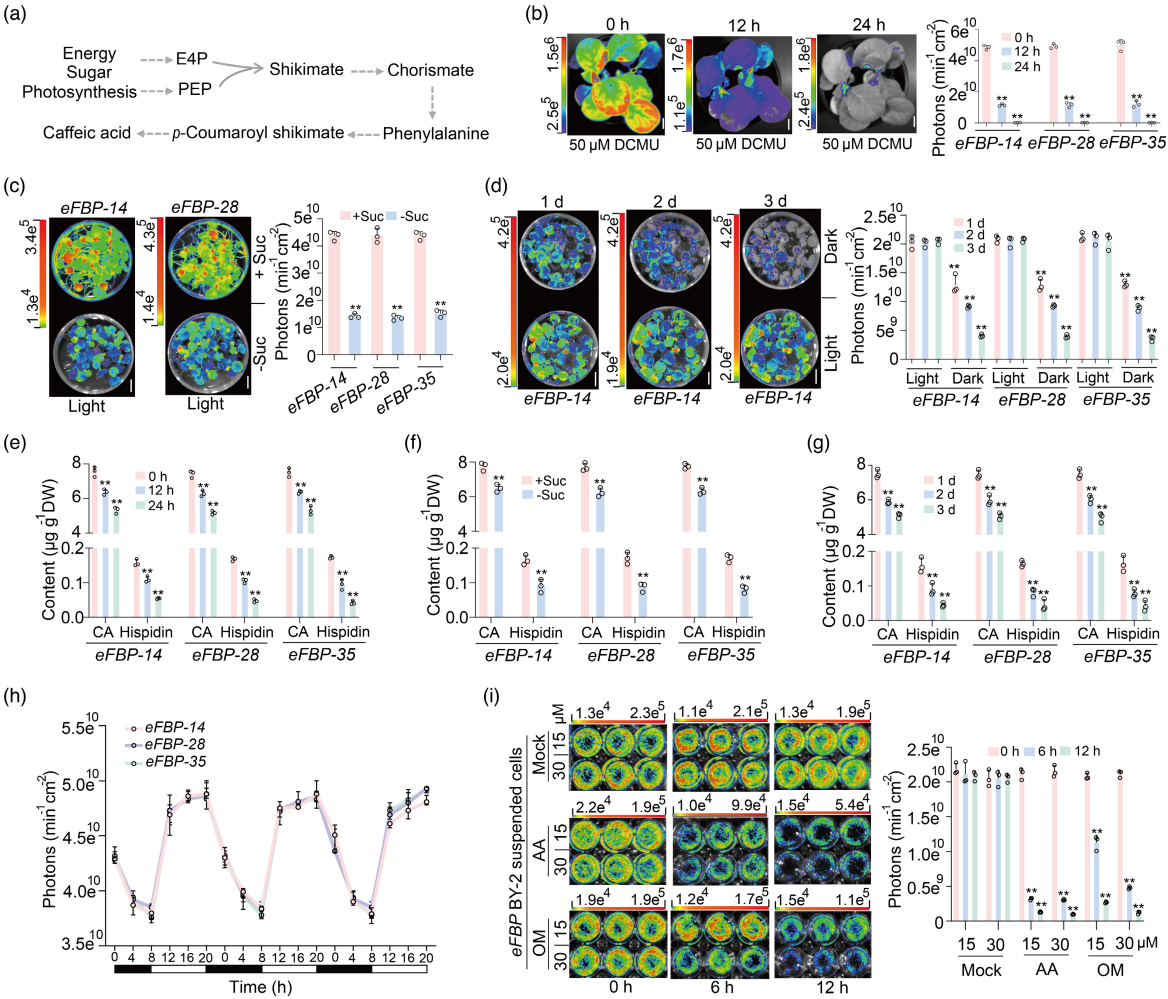
The *eFBP* system is energy dependent in plants. (a) The proposed caffeic acid biosynthetic network derived from sugar. (b) Photosynthesis inhibitor herbicide 3‐(3,4‐dichlorophenyl)‐1,1‐dimethylurea (DCMU) treatment decreases the bioluminescent intensity in *eFBP* transgenic seedlings at 20 DAG. Scale bars, 1 cm. (c) 50 mm sucrose supply boosts bioluminescence emission in 12 DAG seedlings. Growth in plant growth chamber with 12 h Light/12 h Dark condition. Scale bars, 1 cm. (d) The bioluminescent image and statistical analysis of photons emission from *eFBP* transgenic seedlings under continuous dark or light for 3 days. (e–g) LC–MS/MS analysis of caffeic acid and hispidin contents in leaves from *FBP* and *eFBP* transgenic seedlings with DCMU treatment (e), sucrose supply (f), light and dark conditions (g). Error bars indicate means ± SD (*n* = 3). Statistical significance was assessed using two‐tailed *t*‐tests (*P ≤ 0.05, **P ≤ 0.01). (h) Diurnal bioluminescence patterns of *eFBP* transgenic seedlings under 12 h light/12 h dark growth chamber. The open and filled bars at the bottom represent the light and dark periods, respectively. (i) Energy produce inhibitors reduce bioluminescence signal in *eFBP* transgenic tobacco suspended BY‐2 cells. antimycin A (AA), oligomycin (OM). Error bars indicate means ± SD (*n* = 3). Statistical significance was assessed using two‐tailed *t*‐tests (*P ≤ 0.05, **P ≤ 0.01).

Energy including sugar gained from photosynthesis is vital to biosynthetic processes. The biosynthesis of caffeic acid and hispidin in plants mainly starts with the aromatic amino acid phenylalanine derived from shikimate pathway, which converts phosphoenolpyruvate (PEP) and erythrose 4‐phosphate (E4P) from glycolysis and the pentose phosphate pathways (PPP) respectively into chorismate, the final common precursor of all phenylpropanoids (Lee *et al*., 2010; Maeda and Dudareva, 2012; Vogt, 2010). Based on the knowledge of phenylpropanoid and shikimate biosynthesis (Figure 4a), we investigated the necessity of energy in the eFBP pathway using the herbicide 3‐(3,4‐dichlorophenyl)‐1,1‐dimethylurea (DCMU) to inhibit photosynthesis (Calvayrac *et al*., 1979; Xiong *et al*., 2013) of *eFBP* transgenic plants, and the luminescence signal decreased sharply after DCMU treatment (Figure 4b). In plants, photosynthesis converts photons of light through photosynthetic CO_2_ fixation into sugars (Gibbs, 1967; Heyduk *et al*., 2019). So we transferred 6‐day *eFBP* plants to the medium with and without 50 mm sucrose supply for 5 days under 12 h light/12 h dark condition, the plants with sucrose supply emitted around 2.9 times the amount of photons than plants with no sucrose added (Figure 4c). The data strongly supported the fact that sugars strengthened luminescence signal from *eFBP* plants. As light is the plant's source of energy harnessed through photosynthesis, we moved non‐sucrose treated *eFBP* plants mentioned above to continuous dark treatment, the luminescence signal decreased gradually under dark conditions compared with plants under 12 h light/12 h dark conditions (Figure 4d). Then, we used LC–MS/MS analysis to evaluate whether the reduced luminescence signal was due to decreased accumulation of luminescent precursors, caffeic acid and hispidin. The data clearly showed that DCMU treatment, lacking sucrose supply or extended darkness significantly reduced the biosynthesis of caffeic acid and hispidin in *eFBP* plants (Figure 4e–g). These findings provided a mechanistic explanation that luminescent precursors derived from sugars, which are generated from photosynthesis in *eFBP* plants.

The energy status of plants is reflected directly by the availability of energy‐rich products such as ATP, which was carried out via biosynthesis in mitochondria and chloroplast thylakoids in plants (Junge and Nelson, 2015; Nelson and Junge, 2015). We tested the luminescence signal from *eFBP* plants growth in non‐sucrose medium under 12 h/12 h diurnal oscillations, the signal showed a significant positive correlation with light (Figure 4h), which further supports the idea that the coupled *FBP* pathway in plants are energy driven. Antimycin A (AA), an inhibitor of the cytochrome pathway in cyclic electron transport in mitochondria and chloroplast, reduces ATP synthesis in plants (Geisler *et al*., 2004). By adding exogenous AA in *eFBP* BY‐2 cell lines, we found that AA inhibited the luminescence signal dramatically (Figure 4i). Similarly, after treatment with oligomycin (OM), an inhibitor of ATP synthase specifically in mitochondrial (Alber and Vanlerberghe, 2021), the *eFBP* BY‐2 cell lines emitted markedly decreased signal (Figure 4i). Taken together, our data firstly revealed endogenous regulation of transgenic *eFBP* plants by energy from sugar or photosynthesis, which will facilitate further metabolic engineering to create stronger autoluminescent plants.

## Discussion

In this report, we demonstrated that plants integrated with the *eFBP* DNA module showed significantly elevated luminescence intensity than the previously reported FBP system in transgenic tobacco plants (Khakhar *et al*., 2020; Mitiouchkina *et al*., 2020). In the pioneering study, although a three‐enzyme (RcTAL, HpaB, HpaC) pathway was built and transiently expressed in an *FBP* (expressing NPGA, Hisps, H3H, Luz and CPH) stable transgenic line to enable enhanced luminescence approaching two times, indicating the boosted tyrosine to caffeic acid pathway was efficiently enhanced plant autoluminescence (Khakhar *et al*., 2020). Whereas, more gene stackings make the vector construction time and labour‐consuming, and probably cause troubles to Agrobacterium‐mediated transgenic procedures and stability in plants. Here, we incorporated the NPGA and BnC3′H1 (Figure 1e) enzymes to achieve luminescence levels approximately three times higher than the FBP pathway (Figure 2b,d), demonstrating the power of the eFBP assembly. Our *eFBP* transgenic BY‐2 cells also revealed that the system is oxygen‐dependent in plants (Figure S13), and that the luminescence signal from detached leaves could continue up to 3 days (Figure S14), exhibiting strong stability and sustainability. Considering that the biosynthetic pathway of caffeic acid in eFBP plants is prone to be disturbed by the ever‐changing surrounding (Figure 3), further metabolic engineering strategy will provide excessive pool of caffeic acid to create stable glowing plants.

**Figure 5:**
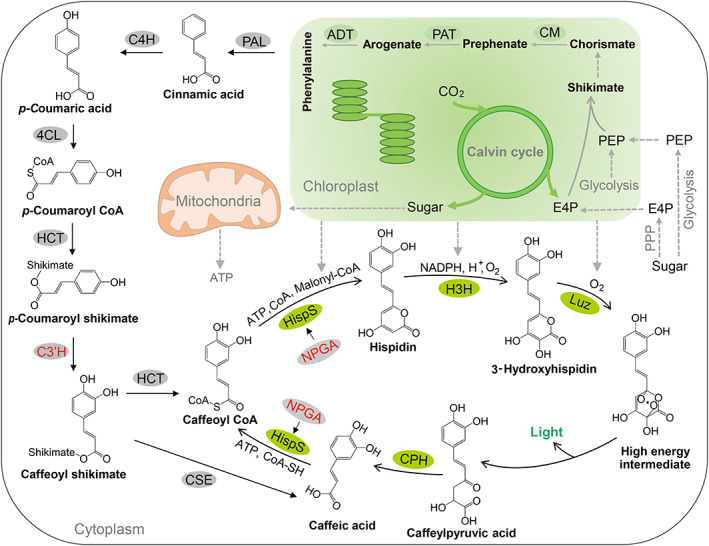
Integration of the FBP into plants' metabolic pathways. Proposed pathway of fungal luciferin biosynthesis and recycling coupled with caffeic acid biosynthesis in photoautotrophic plants. The caffeic acid biosynthesis pathway provides sustained metabolites coupled with bioluminescent cycle. The initial resources and energy originated from carbon assimilation coupled photosynthesis in chloroplast, where phenylalanine is synthesized. E4P, erythrose 4‐phosphate; PEP, phosphoenolpyruvate; CM, chorismate mutase; PAT, prephenate‐aminotransferase; ADT, arogenate dehydratase; PAL, phenylalanine ammonia‐lyase; C4H, cinnamic acid 4‐hydroxylase; 4CL, 4‐hydroxycinnamate:CoA ligase; HCT, 4‐hydroxycinnamoyl CoA:shikimate/quinate hydroxycinnamoyltransferase; C3′H, 4‐coumaroyl shikimate/quinate 3′‐hydroxylase; CSE, caffeoyl shikimate esterase; HispS, hispidin synthase; NPGA, 4′‐phosphopantetheinyl transferase; H3H, hispidin‐3‐hydroxylase; Luz, luciferase; CPH, caffeylpyruvate hydrolase; ATP, Adenosine triphosphate; CoA, Co‐enzyme A; NADPH, Nicotinamide adenine dinucleotide phosphate.

Life is centred around the production and utilization of photosynthetic product sugars that serve as the primary supplies of energy and building blocks in plants, also the bioluminescence requires energy in plants. Significantly, we creatively present that the eFBP system is energy‐driven in plants (Figure 4). The new finding indicates that further engineering of FBP system in plants or animals should systematically consider different factors that may affect the coupled FBP system, including energy supply and surrounding issues. Photoautotrophic plants possess a unique advantage in moving toward green energy for producing all substrates and co‐factors essential for the optimal operation of the FBP system, including CoA, Malonyl‐CoA, NADPH, ATP, proton and oxygen through photosynthesis and respiration (Bailey‐Serres *et al*., 2018; Griffin and Heskel, 2013; Heyduk *et al*., 2019; Pan *et al*., 2020) (Figure 5). This study laid the groundwork for developing biological reporting tools in plant science, and highlights the inherent coupling between the FBP and the plant's metabolism which should be considered when designing biosensors, which also inspire us to engineer brighter horticulture plant for decoration or illumination in rural areas, these plants could be the potential green energy resource with reduced carbon footprint.

Importantly, the luminescence precursor substrates caffeic acid and hispidin are water‐soluble, cell absorbable and low toxic in plant and animal cells (Kotlobay *et al*., 2018; Reuter *et al*., 2020). The system could be explored in various applications in molecular biology and biosensors for the environment, biosecurity and health purposes. Powerful unbiased mutagenesis, enzyme‐directed evolution and metabolic engineering may help overcome some limitations such as heat sensitivity to achieve further improvement. With continuous optimization of this autoluminescent system, the future would be much brighter.

## Materials and methods

### Structure prediction and molecular docking

The full‐length structure model of C3′Hs was first predicted by AlphaFold2 (Jumper *et al*., 2021). The predicted structures with the highest ranking score were selected for the subsequent molecular dockings of *p*‐coumaroyl shikimate molecule. The 3D structure of *p*‐coumaroyl shikimate was downloaded from ZINC15 (Sterling and Irwin, 2015) (https://zinc15.docking.org/substances/home). The molecular dockings of *p*‐coumaroyl shikimate into the predicted structures of C3′Hs were performed by *AutoDock Vina* 1.2.0 (Trott and Olson, 2010) (https://vina.scripps.edu). The autodockings were performed in the whole space of C3′Hs structure with parameters ‘exhaustiveness = 10’, and ‘num_modes = 20’. The results were presented and analysed in PyMOL (DeLano, 2002), the Top‐5 modes of each docking are shown in Table S1.

### Transient expression for *in vitro* enzyme assays

Target *C3′Hs‐Flag*, *NnHispS‐Flag* and *AnNPGA‐YFP* were cloned into the pCAMBIA1300 vector and transformed into the *Agrobacterium tumefaciens* strain EHA105. Bacteria clones were cultivated in a 50 mL glass flask at 28 °C to OD_600_ of 0.8–1.0, washed with infection buffer (10 mm MgCl_2_, 10 mm MES, 150 μm As, pH = 6), then resuspended the bacteria in infection buffer to OD_600_ of 0.8–1.0, and stranded for 2–3 h before injection. 1 mL of the final culture was used to infiltrate the underside of 5‐week‐old tobacco (*Nicotiana tabacum cv*. Zhongyan 100) leaves. Leaves were harvested 2 day after infiltration for metabolites extraction and measurement by LC–MS/MS.

### Plasmid construction

Coding sequences of the *NnLuz*, *NnHispS*, *NnH3H* and *NnCPH* genes from *N. nambi* and *AnNPGA* gene from *A. nidulans* were codon optimized for expression in *N. tabacum* and ordered synthetically from Shanghai Generay Biotech Co., Ltd. The coding sequence of the *BnC3′H1* was amplified from *Brassica napus cv*. Westar. To assemble multiple target genes into a single plant transformation vector, donor vectors with the above six genes expression cassettes under the control of the constitutive 35S promoter from cauliflower mosaic virus were constructed by traditional restriction ligation method, using pYL322d1 or pYL322d2 as vector backbones based on previous report (Zhu *et al*., 2017). The 35S promoter (P35S) and NOS terminator (Tnos) were ligated into the pYL322d1 plasmid via XhoI/XbaI and SalI/HindIII, respectively, to produce pYL322d1‐P35S. While pYL322d2‐P35S was obtained by ligating the P35S and Tnos into the pYL322d2 plasmid via HindIII/NcoI and EcoRI/XhoI, respectively. *NnHispS*, *NnH3H* and *NnLuz* were cloned into pYL322d1‐P35S via EcoRI and SalI sites, and *NnCPH*, *AnNPGA* and *BnC3′H1* were ligated into the BamHI and EcoRI sites of pYL322d2‐P35S.

The TransGene Stacking II (TGSII) system was used to realize the assembly of multiple genes (Zhu *et al*., 2017). Following the detailed protocol of TGSII, *NnHispS*, *NnCPH*, *NnH3H*, *AnNPGA*, *NnLuz* and *BnC3′H1* were sequentially delivered into the binary vector pYLTAC380GW to obtain 380GW‐35S‐4G, 380GW‐35S‐5G and 380GW‐35S‐6G constructs. Finally, the selectable marker/marker‐excision cassette in marker‐free donor vector pYLMF‐H, in which anther‐specific promoter P_AS_ from *N. tabacum*, was recombined into above 380GW constructs by Gateway BP reaction according to the protocol for the Gateway BP Clonase II Enzyme Mix kit (catalogue no. 11789–020, Invitrogen) to produce 380MF‐35S‐6G, 380MF‐35S‐7G and 380MF‐35S‐8G harbouring *oFBP*, *FBP* and *eFBP* modules, respectively. The detailed vectors are shown in Extended Table 2. The resulting constructs were analysed and identified by Not I digestion, and correct sequences of all plasmids were confirmed with Sanger and Illumina sequencing before use. All primers used in the plasmid constructions are shown in Table S3.

### Agrobacterium‐mediated plant transformation

Assembled plasmids were transferred into *Agrobacterium tumefaciens* strain EHA105. Bacteria were cultured in LB liquid medium supplemented with 25 mg/L rifampicin and 50 mg/L kanamycin sulphate in flasks shaken at 200 rpm at 28 °C to an OD_600_ of 0.8. Bacteria were collected by centrifugation, and then resuspended in liquid MS_0_ medium (MS salts, 30 g/L sucrose, 0.15 mm acetosyringone (As), pH 5.8) to an OD_600_ of 0.6 (for tobacco and poplar transformation) or 0.25 (for BY‐2 cell transformation). The bacterial suspensions were incubated for 2 h at room temperature without shaking before using.

For tobacco transformation, fully expanded leaves were harvested from 4‐week‐old tobacco plants. Removing the leaf midrib and the leaf edge with a scalpel, and then cutting the lamina into small pieces approximately 0.5 cm^2^. Place the explants adaxial‐side up on a MS_1_ medium (MS salts, 30 g/L sucrose, 0.5 mg/L IAA, 2 mg/L 6‐benzylaminopurine (6‐BA), 6.5 g/L phytagel, pH 5.8) for 2 day under constant dark at 25 °C. Precultured leaf explants were incubated with bacterial culture for 20 min, and then placed onto filter paper overlaid on MS1 medium. Two days after inoculation in the dark, explants were transferred to the same medium supplemented with 400 mg/L timentin and 50 mg/L hygromycin B. Regeneration shoots were cut and grown on MS medium with antibiotics.

For poplar transformation, tissue‐cultured hybrid poplar (*Populus alba* × *Populus glandulosa*, Pag) 84 K plants were grown under long‐day conditions (16 h light/8 h dark). Leaf discs were used for Agrobacterium‐mediated transformation as described previously (Zhou *et al*., 2019). Leaf discs were incubated with Agrobacterium harbouring relative constructs for 10 min and then cultured in darkness for 3 days on shoot‐induction medium (MS basal medium containing 0.5 mg/L 6‐BA and 0.05 mg/L naphthalene acetic acid (NAA)). After cultivation for ~30 days on shoot‐induction medium supplemented with 3 mg/L hygromycin and 200 mg/L Timentin, regenerated shoots were selected and transferred to root‐induction medium (1/2 MS medium supplemented with 0.05 mg/L IAA and 0.02 mg/L NAA). Transgenic plants were confirmed by photographic instrument and qPCR assay.

For BY‐2 cell transformation, 7‐day‐old BY‐2 cells suspension was mixed with agrobacterium culture and incubated at 130 rpm at 26 °C in the dark. After 2 days incubation, cells were spun down at 200 *g* for 2 min. Pellet was washed with MS_1_ medium (4.3 g/L MS salts, 30 g/L sucrose, 0.255 g/L KH_2_PO_4_, vitamin, pH 5.0) for 3 times, and then transferred to MS_2_ selective plates (MS_1_ medium supplemented with 50 mg/L hygromycin, 200 mg/L Timentin and 7 g/L Agar, pH 5.8) containing filter paper and spread out evenly over the filter paper. After 2 weeks of selection on MS_2_ plates, bright yellow calli in good condition were transferred onto new MS_2_ selective plates for further selection. Positive transgenic BY‐2 cells were identified by photographic instrument and used for the study.

### Gene expression analysis

All leaves were flash frozen in liquid nitrogen and homogenized for RNA extraction with TRIzol reagent (Invitrogen). The first‐stranded cDNA was synthesized from 1 μg of RNA using a MonScriptTM RTIII Super Mix with dsDNase (Two‐Step) (Monad Biotech, China) following the manufacturer's instruction. For RT‐qPCR, gene transcript levels were quantified using SYBR Premix with gene‐specific primers on a LightCycler480 II Real‐Time PCR machine (Roche) with the following program: 95 °C for 1 min and then 40 cycles of 95 °C for 10 s, 60 °C for 20 s and 72 °C for 20 s. For each sample, at least three biological replicates were analysed. *NtEF1a* was used as reference genes for normalizing gene expression, and all primers are shown in Table S3.

### Growth conditions and stresses treatments

To examine the transcript levels of *NtCMs*, *NtPAT*, *NtADTs*, *NtPALs*, *NtC4H*, *Nt4CL*, *NtHCT*, *NtCSE* and *NtC3′Hs* under various stresses, seeds of transgenic tobacco plants were germinated on Murashige and Skoog (MS) medium in a growth chamber with 14 h light/10 h dark cycle for 7 days, and then the seedlings were transplanted into pots. For cold and heat stresses, tobacco at the 15 days were transferred to a growth chamber at 4 °C (cold stress) or 37 °C (heat stress) and sampled at 0, 0.2, 1.5 and 12 h after the treatment. For drought stress, watering was stopped and leaves were sampled at the following time points: control, no stress; day 1 (when seedlings showed slight leaf wilting); day 2 (the second day after time point day 1); day 3 (the third day after time point day 1). For UV light stress, seedlings were transferred to a tissue culture room with UV light emission peak at 254 nm, 1100 μW/cm^2^ at plant level, sampled at 0, 1.5, 3 and 12 h. All samples were frozen immediately in liquid nitrogen.

### LC–MS/MS analysis

The harvested plant samples were immediately frozen in liquid nitrogen followed by lyophilization in 50 mL Falcon tubes. About 100 mg of the lyophilized powder was measured and transferred to 5 mL extraction buffer containing 70% methanol. The extracts were ultrasonicated in a water bath for 30 min, followed by centrifugation at 13 000 ***g*** for 15 min. The supernatants were then filtered through a PVDF syringe filter (pore size 0.45 μm) and transferred to glass vials for LC/MS analysis. The samples were run on an Exion HPLC system coupled to an AB SCIEX QTRAP 6500plus mass spectrometer. Five microliters of the samples were injected into an ACQUITY BEH C18 column (2.1 × 50 mm). The liquid chromatographic separation method used the following mobile phase at a flow rate of 0.4 mL/min: 0.1% formic acid as solvent A and 100% acetonitrile as solvent B. The LC separation was performed using an 8‐min linear elution gradient: 5% B at 0 min, 20% B at 1.5 min, 50% B at 3.5 min, 90% B at 5 min, and held at 90% B until 6.5 min, return to 5% B at 6.6 min and held at 5% B until 8 min.

The MS was performed under negative ion‐mode electrospray ionization (ESI‐). Quantification of target metabolites was carried out using multiple reaction monitoring (MRM) modes with the following settings: curtain gas, 35 psi, ion spray Voltage −4.5 kV, ion source temperature, 500 °C, ion source gas 1, 55 psi, ion source gas 2, 50 psi. The MRM transitions of targeted metabolites were 179.0 and 135.0 for caffeic acid, 158.0 and 126.0 for *p*‐Coumaroyl shikimate, and 245.0 and 159.1 for hispidin. The declustering potential (DP), collision energy (CE), and cell exit potential (CXP) were set at 80 V, 20 V, 10 V for caffeic acid, and 85 V, 20 V and 7 V for *p*‐Coumaroyl shikimate, 98 V, 27 V, 10 V for hispidin. The dwell time was set at 100 ms. Analytical standards of hispidin were purchased from Sigma‐Aldrich. Caffeic acid and *p*‐Coumaroyl shikimate from Sangon Biotech, and A series of analytical standard dilutions were used for setting up the standard curve and calculating the concentration of targeted metabolites. All data were collected and processed using the Analyst 1.6.3 Software.

### Western blot analysis

Tobacco leaves transiently expressing fusion proteins were homogenized in liquid nitrogen using a mortar and pestle. Each 0.1 g sample of homogenized leaves was combined with 200 μL extraction buffer (50 mm Tris–HCl, pH 7.5, 150 mm NaCl, 0.5% TritonX‐100, Roche cocktail protease inhibitor) and centrifuged at 13500 *g* at 4 °C for 10 min. The supernatant was transferred to a new tube, and one‐third volume of 4× Laemmli buffer (250 mm Tris–HCl, pH 6.8, 8% SDS, 40% glycerol, 4% β‐mercaptoethanol, 0.01% bromophenol blue) was mixed with the supernatant. The mixture was denatured at 95–100 °C for 10 min. Total proteins were separated by sodium dodecyl sulphate‐PAGE (SDS‐PAGE), transferred onto a polyvinylidene difluoride membrane (Millipore) and incubated with various primary antibodies against tags Flag or YFP. Actin antibody was used as loading control. Detection was carried out using ECL Western Blotting Detection Reagents (Bio‐Rad).

### Plant imaging

Plant bioluminescence signal imaging and photons dose calculations are performed on the NIGHTSHADE LB985, an instrument made in Germany whose core component is a back‐through ultra‐sensitive CCD camera. The samples were placed internally at a height of about 40 cm from the top of the completely light‐protected dark box. The bioluminescent images were taken with 60‐s exposure, and then regions for calculating the photons were selected. Finally, data was exported for analysis. Ambient light images were taken after the luminescence measurements. Other settings were left at defaults.

We used a Nikon D750 camera with AF‐S17‐35 mm F2.8D ED‐IF at ISO 2000, F6.3, 60‐s shutter speed to capture dark condition photos of Figure 2a,e,f, Figures S7a, S10a,b, S13. Video S1 was taken with Sony Alpha 1, Sony GM 50 mm f1.2, 1/30 f1.2 iso32000.

### Statistics

Most of the quantitative data shown in this paper represent mean ± s.d. from at least three biological replicates. Statistical analyses were performed with GraphPad Prism 9 software. Statistical significance was assessed using an unpaired two‐tailed Student's *t*‐test when comparing two groups. The exact *P* values are indicated in the figures.

## Conflict of interest

The authors declare no competing interests.

## Author contributions

P.Z. and H.D. initiated the project and designed the experiments, P.Z., H.D., J.G., J.J., J.Z., B.B., W.L., Y.M., D.L., W.T., H.C. and Y.Z. performed experiments. S.M. provided information of tobacco genes. Y.Z., J.W. and B.B performed bioinformatics analyses. L.H. and P.F. conducted LC–MS/MS data acquisition and analysis. P.Z. and H.D. wrote the manuscript. All authors discussed the results and commented on the manuscript.

## Supporting information


**Figure S1** Protein sequences cluster of C3′H homologues.
**Figure S2** Transiently expressing C3′H1 constructs for enzyme activity assay.
**Figure S3** Molecular modelling of BnC3′H1.
**Figure S4** Multiple sequence alignment of C3′H homologues.
**Figure S5** Identification of the *FBP* and *eFBP* DNA modules and transgenic tobacco lines.
**Figure S6** Identification of the *FBP* and *eFBP* transgenic tobacco lines.
**Figure S7** *FBP* and *eFBP* transgenic lines at the flowering stage.
**Figure S8** Characterization of selectable marker excised plants from *eFBP* transgenic lines.
**Figure S9** Analysis of the light emission from *FBP* and *eFBP* BY‐2 cell lines.
**Figure S10** The test of *eFBP* module to generate luminescence in diverse plant species by transient expression.
**Figure S11** Identification of *eFBP* transgenic poplar lines.
**Figure S12** Analysis of the stability of *eFBP* transgenic tobacco to abiotic stresses.
**Figure S13** Oxygen requirement for bioluminescent in *eFBP* transgenic BY‐2 cells.
**Figure S14** The stability of photon emission from detached leaves of *eFBP* transgenic tobacco seedlings.
**Video S1** The video shows immediate visualization of the auto‐illumination plants in dark room.
**Table S1** The molecular dockings of *p*‐Coumaroyl shikimate into the predicted structure of C3'Hs.
**Table S2** Vectors used in this study.
**Table S3** Primers used in this study.
