# Supplementary material for: Metabolic engineering and mechanical investigation of enhanced plant autoluminescence
Source: Plant Biotechnol J. 2023 May 8;21(8):1671–81. doi: 10.1111/pbi.14068 (PMC10363767; doi:10.1111/pbi.14068)
Supplement: Supplementary file 1 — Figure S1 Protein sequences cluster of C3′H homologues. Figure S2 Transiently expressing C3′H1 constructs for enzyme activity assay. Figure S3 Molecular modelling of BnC3′H1. Figure S4 Multiple sequence alignment of C3′H homologues. Figure S5 Identification of the FBP and eFBP DNA modules and transgenic tobacco lines. Figure S6 Identification of the FBP and eFBP transgenic tobacco lines. Figure S7 FBP and eFBP transgenic lines at the flowering stage. Figure S8 Characterization of selectable marker excised plants from eFBP transgenic lines. Figure S9 Analysis of the light emission from FBP and eFBP BY‐2 cell lines. Figure S10 The test of eFBP module to generate luminescence in diverse plant species by transient expression. Figure S11 Identification of eFBP transgenic poplar lines. Figure S12 Analysis of the stability of eFBP transgenic tobacco to abiotic stresses. Figure S13 Oxygen requirement for bioluminescent in eFBP transgenic BY‐2 cells. Figure S14 The stability of photon emission from detached leaves of eFBP transgenic tobacco seedlings. Video S1 The video shows immediate visualization of the auto‐illumination plants in dark room. Table S1 The molecular dockings of p‐Coumaroyl shikimate into the predicted structure of C3'Hs. Table S2 Vectors used in this study. Table S3 Primers used in this study. [file PBI-21-1671-s001.zip › pbi14068-sup-0001-Supinfo.zip.pdf]

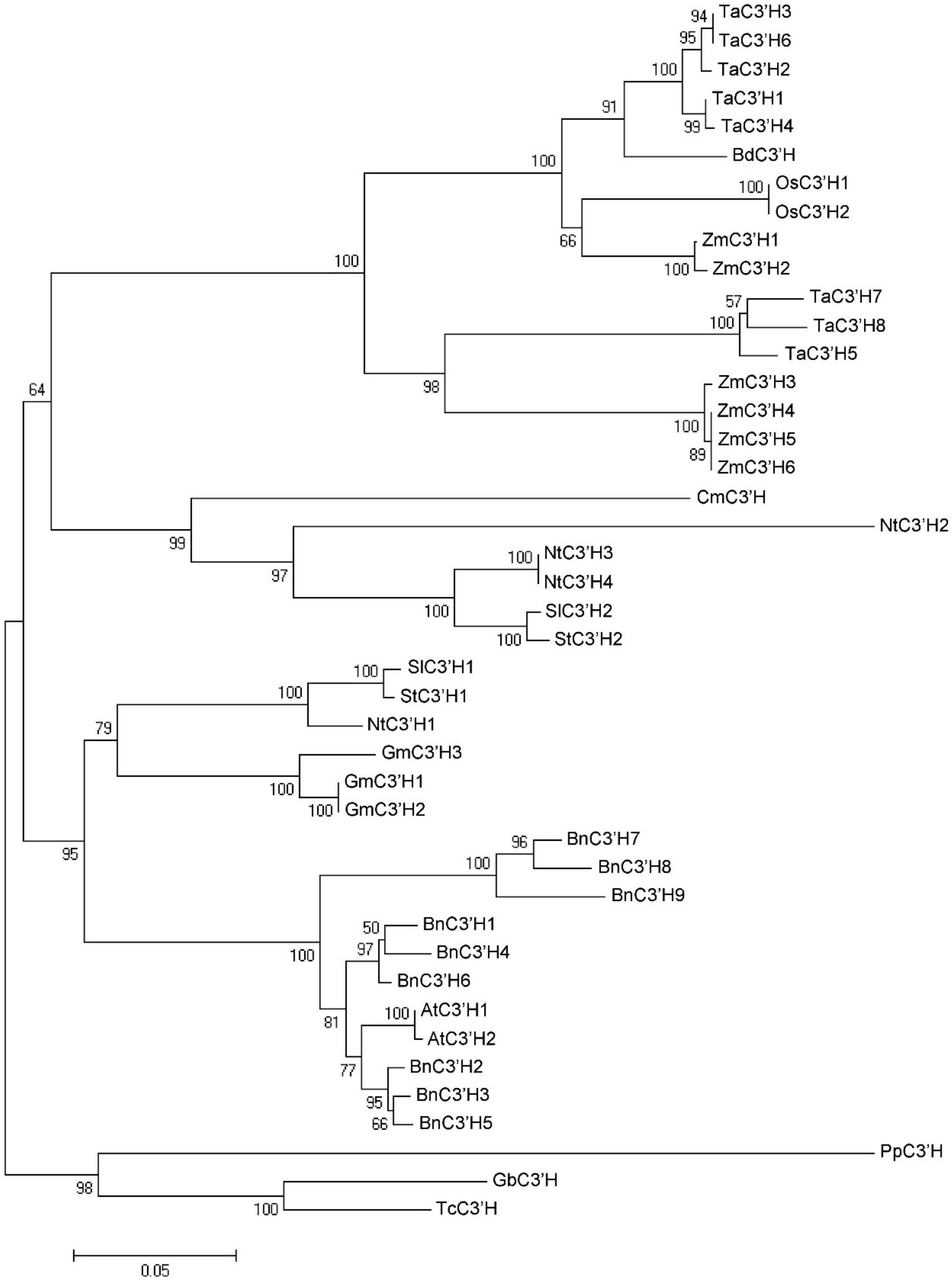

Figure S1 Protein sequences cluster of C3'H homologs.

The full-length amino acid sequence of 43 C3'Hs from 14 plants species were retrieved with BLASTP (http://www.ncbi.nlm.nih.gov/), including *Arabidopsis thaliana* (AtC3'H1: NP_850337.1; AtC3'H2: BAE98524.1), *Brassica napus* (BnC3'H1: XP_013727380.2; BnC3'H2: XP_013734498.1; BnC3'H3: XP_013647851.2;

BnC3'H4: XP_013647763.2; BnC3'H5: CAF1700952.1; BnC3'H6: CAF1870414.1; BnC3'H7: CAF2093614.1; BnC3'H8: XP_013681641.3; BnC3'H9: XP_048610387.1), *Oryza sativa* (OsC3'H1: XP_015639951.1; OsC3'H2: AAU44038.1), *Zea mays* (ZmC3'H1: NP_001142110.1; ZmC3'H2: ACG25686.1; ZmC3'H3: PWZ32976.1; ZmC3'H4: NP_001130442.1; ZmC3'H5: ACG39178.1; ZmC3'H6: XP_0018672021.1), *Nicotiana tabacum* (NtC3'H1: XP_016482834.1; NtC3'H2: XP_016442382.1; NtC3'H3: ABC69384.1; NtC3'H4: XP_016479719.1), *Glycine max* (GmC3'H1: XP_003521103.1; GmC3'H2: KAH1069646.1; GmC3'H3: NP_001235563.1), *Triticum aestivum* (TaC3'H1: XP_044339541.1; TaC3'H2: XP_044413634.1; TaC3'H3: XP_044450947.1; TaC3'H4: CAE47490.1; TaC3'H5: XP_044350510.1; TaC3'H6: CAE47489.1; TaC3'H7: XP_044341732.1; TaC3'H8: XP_044358178.1), *Physcomitrium patens* (PpC3'H: XP_024360823.1), *Ginkgo biloba* (GbC3'H: AAY54293.1), *Taxus chinensis* (TcC3'H: KAH9304895.1), *Brachypodium distachyon* (BdC3'H: XP_003568200.1), *Solanum lycopersicum* (SlC3'H1: XP_004230046.1; SlC3'H2: XP_004249116.1), *Solanum tuberosum* (StC3'H1: XP_006347680.1; StC3'H2: XP_006364777.1), and *Chrysanthemum x morifolium* (CmC3'H: QBA30069.1). Sequence alignment was done using ClustalX 2.0(Larkin et al., 2007), and the resultant alignment was used to construct an NJ tree with MEGA 5.0(Tamura et al., 2011), using Poisson correction, pairwise deletion, and 1000 bootstrap replicates.

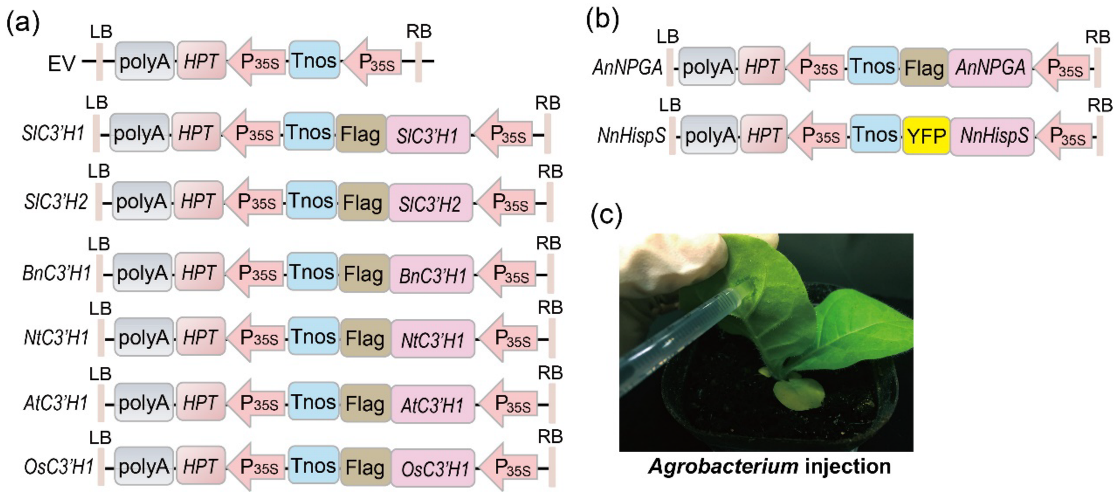

Figure S2 Transiently expressing C3'H1 constructs for enzyme activity assay.

(a, b) Schematic diagram of the expression constructs. LB, Left border; RB, right border.
(c) Injection in *Nicotiana tabacum* leaves for transiently expressing indicated constructs.

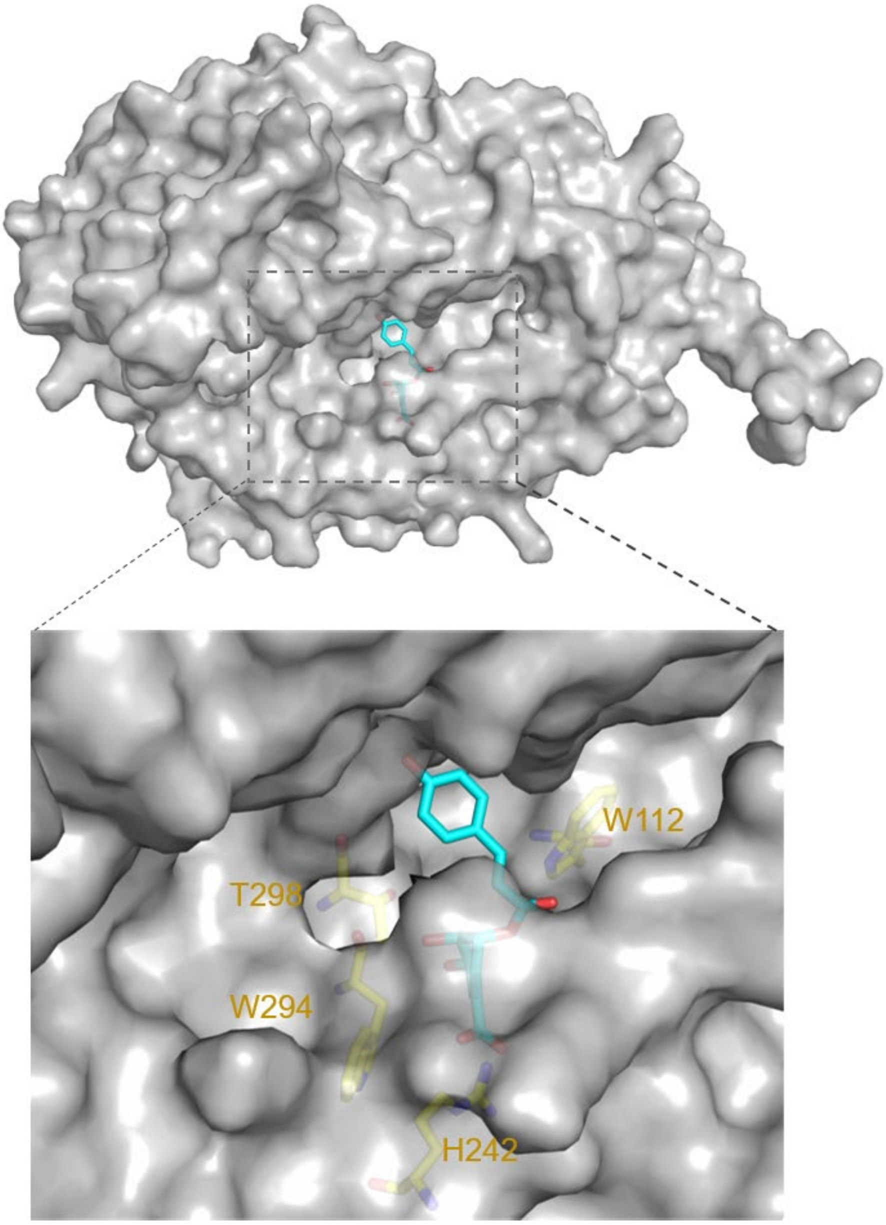

Figure S3 Molecular modeling of BnC3'H1.

The holo-crystal structure of BnC3'H1 in complex with *p*-coumaroyl shikimate. The substrate *p*-coumaroyl shikimate was computationally docked into the active site. A detailed view of the BnC3'H1 active site, highlighting several predicted key residues involved in substrate binding and catalysis.

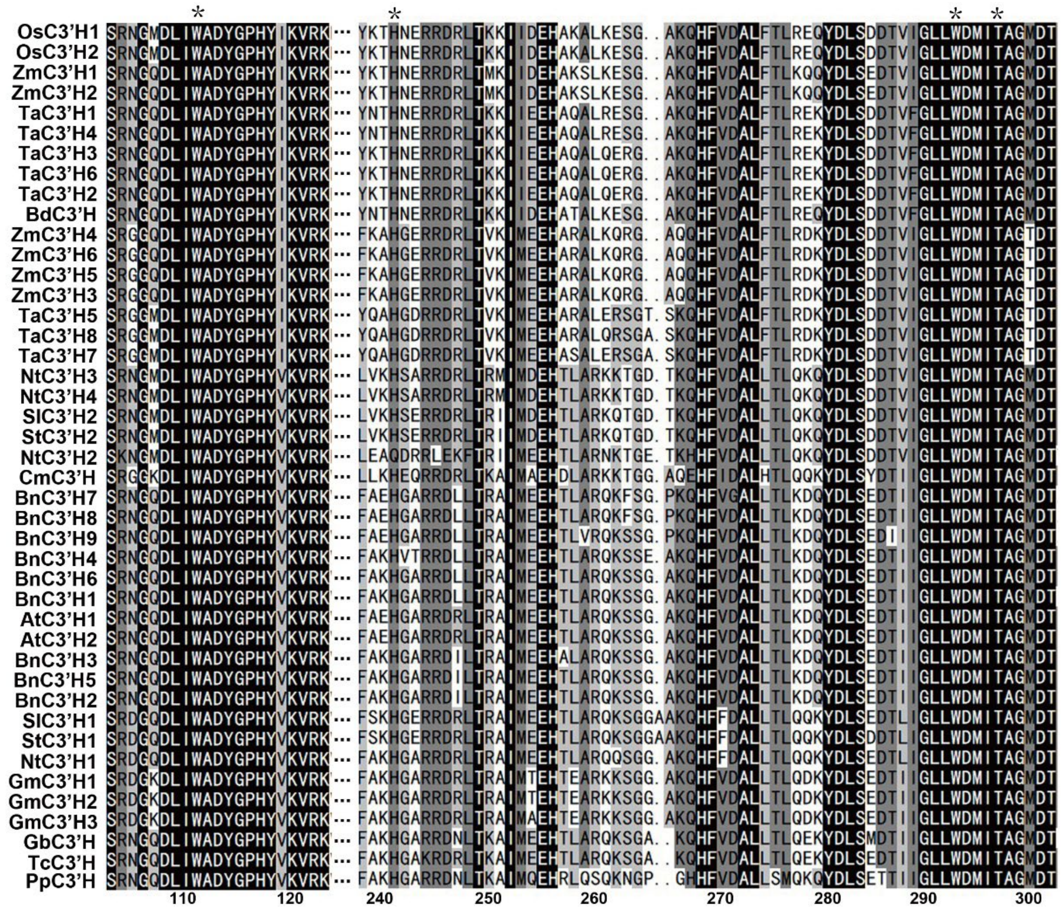

Figure S4 Multiple sequence alignment of C3’H homologs.

Sequence alignment was done using ClustalX 2.0. Only sequences around residues W112, H242, W294, and T298 indicated by stars were shown.

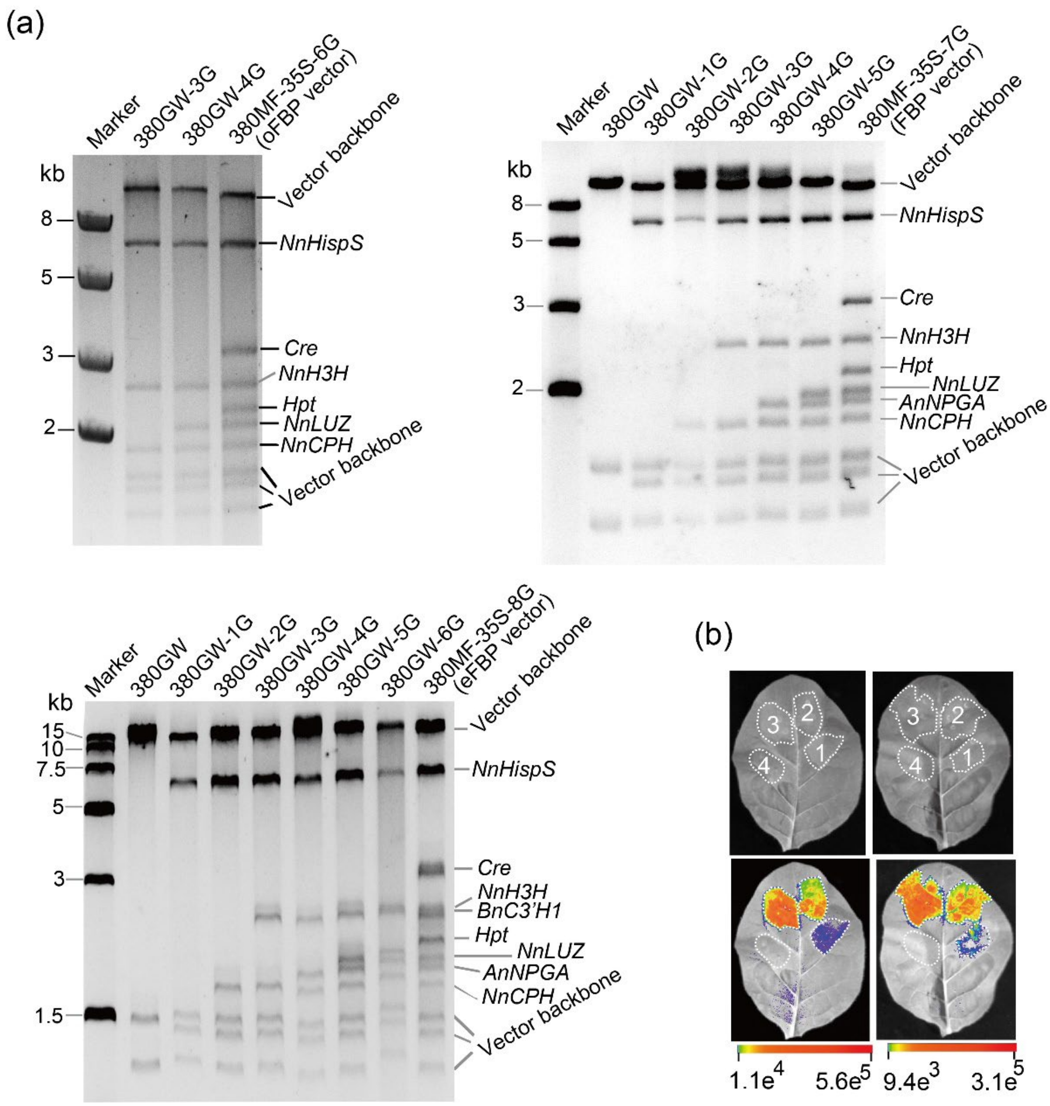

Figure S5 Identification of the *FBP* and *eFBP* DNA modules and transgenic tobacco lines.

(a) Not I-restriction analysis of the acceptor constructs from different rounds of gene assembly with increasing numbers of target genes. For structural stability testing of pYLTAC380MF-6G (oFBP), pYLTAC380MF-7G (FBP) and pYLTAC380MF-8G (eFBP) in the *A. tumefaciens* strain EHA105, the plasmid was introduced into EHA105, isolated from colonies, and transferred back into *E. coli*, and isolated for restriction analysis.

(b) The test of (1) *oFBP*, (2) *FBP* and (3) *eFBP* modules to generate luminescence in *Nicotiana tabacum* leaves by transient expression. (4) *EV*, empty vector.

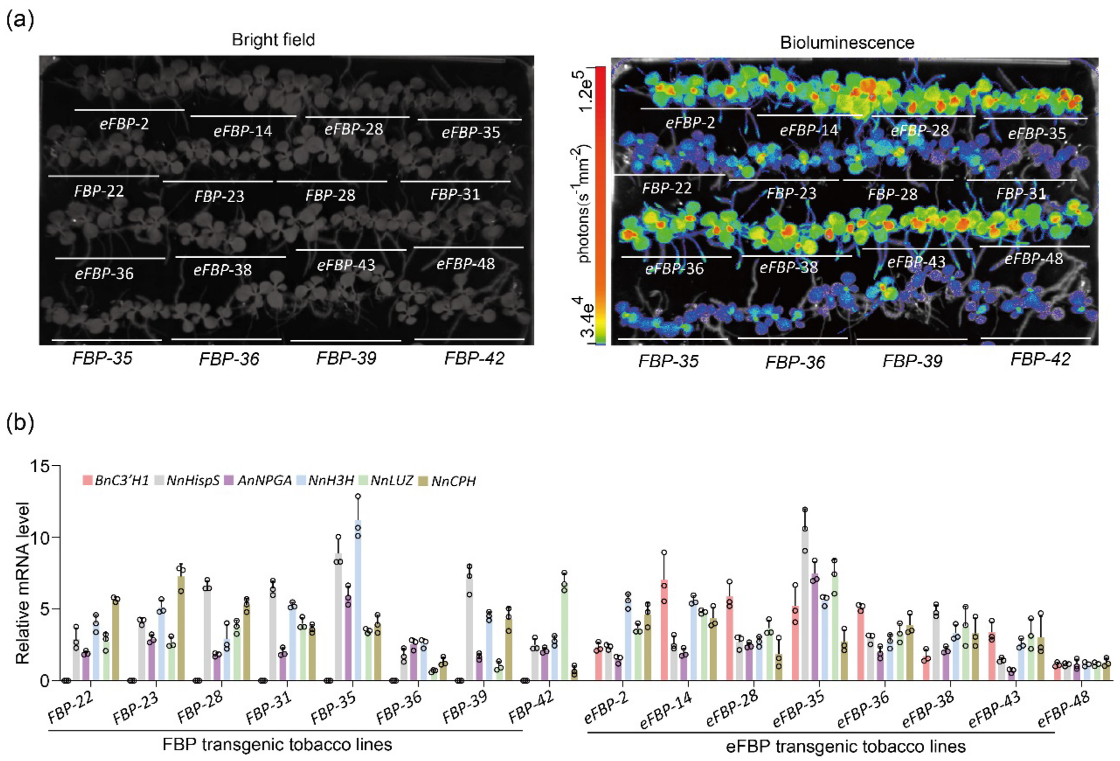

Figure S6 Identification of the *FBP* and *eFBP* transgenic tobacco lines.

(a) Screen of independent *FBP* and *eFBP* transgenic T2 generation lines by photographic instrument.

(b) RT-qPCR assay of the stronger independent *FBP* and *eFBP* transgenic lines after preliminary screen.

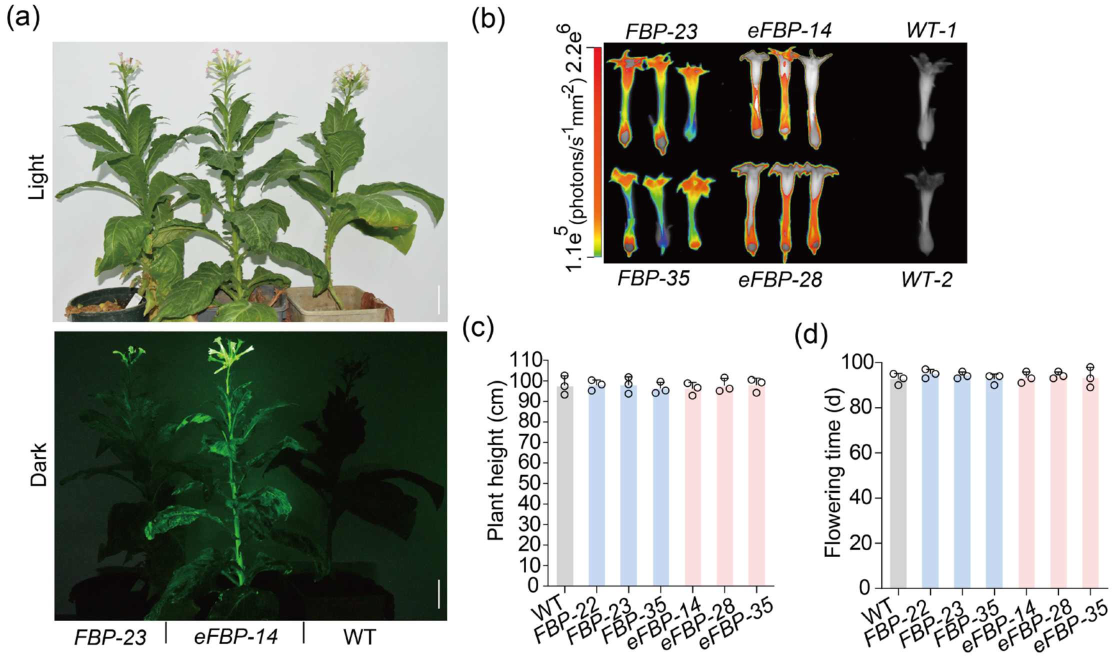

Figure S7 *FBP* and *eFBP* transgenic lines at the flowering stage.

(a) Photos were taken in ambient light with 1/200 sec exposure and in the dark with 60-sec exposure, respectively. Glowing plants were captured with a Nikon D750 camera and a Nikon AF-S17-35mm F2.8D ED-IF at ISO 2000, F6.3, and 60-sec shutter speed. Scale bars, 10 cm.

(b) Identification of the bioluminescent intensity from flowers of *FBP* and *eFBP* lines at 80 DAG.

(c, d) Statistical data of plant height (c) and flowering time (d) of transgenic tobacco in greenhouse. Values are mean ± SD (n = 3).

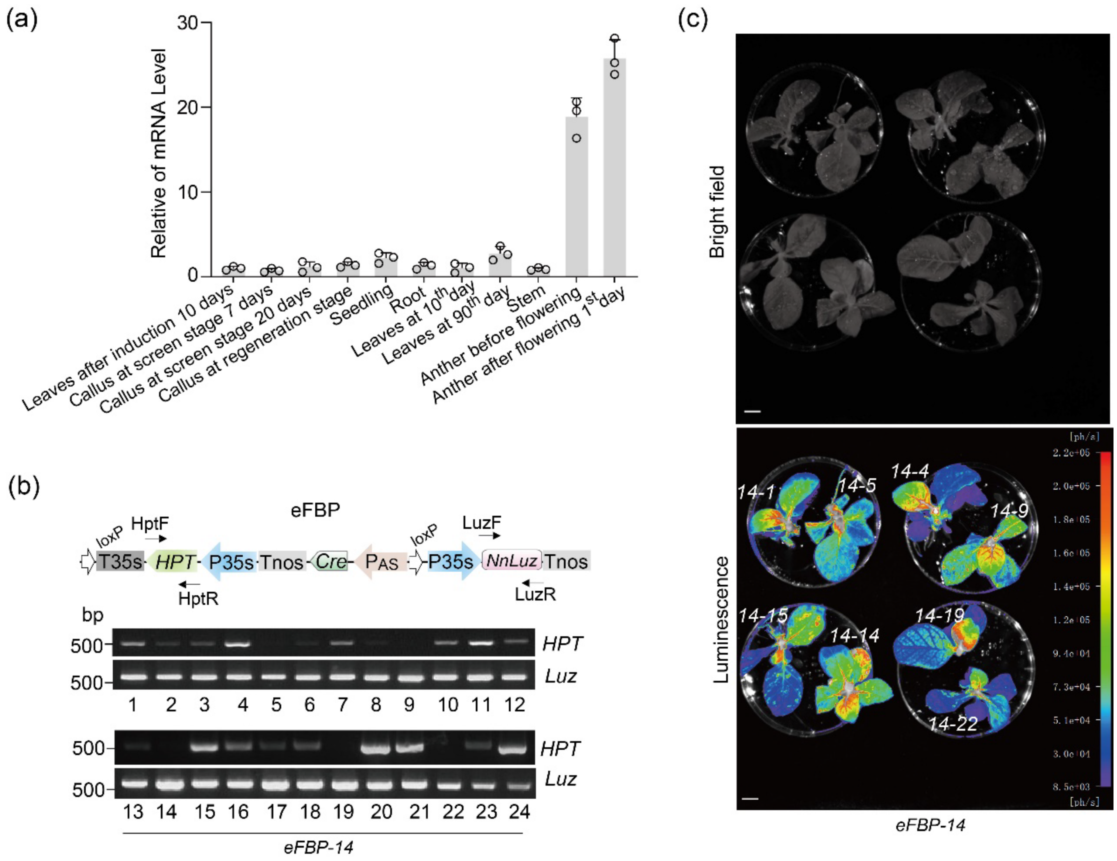

Figure S8 Characterization of selectable marker excised plants from *eFBP* transgenic lines.

(a) Identification of an anther specific promoter in tobacco by RT-qPCR.

(b) PCR analysis of the T3 *eFBP* transgenic tobacco genomic DNAs with specific primers, five T3 *eFBP* transgenic tobacco plants 14-5, 14-9, 14-14, 14-19, 14-22 are selectable marker free glowing plants.

(c) Detection of selectable marker-free glowing plants by photographic instrument.

Scale bars, 1 cm.

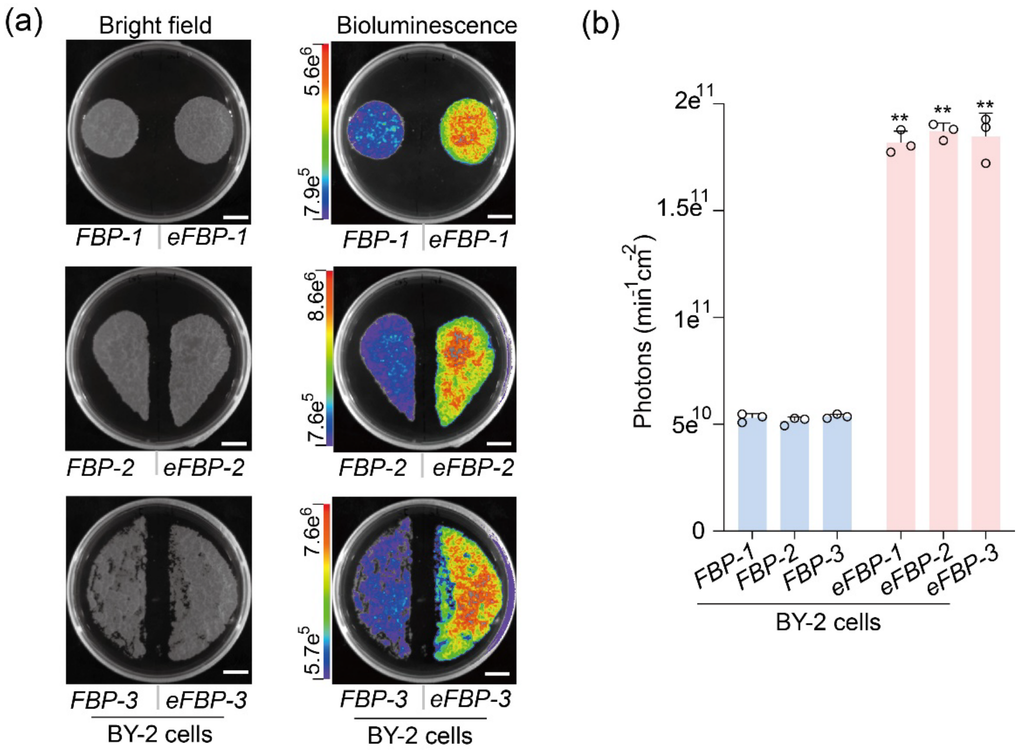

Figure S9 Analysis of the light emission from *FBP* and *eFBP* BY-2 cell lines.

(a) Total photon flux analysis of light emission from three independent *FBP* and *eFBP* transgenic BY-2 cell lines.

(b) The average radiance of photon flux of light emission from Figure S8a. Error bars indicate means ± SD (n=3). Statistical significance was assessed using two-tailed t-tests (**P ≤ 0.01). Scale bars, 1 cm.

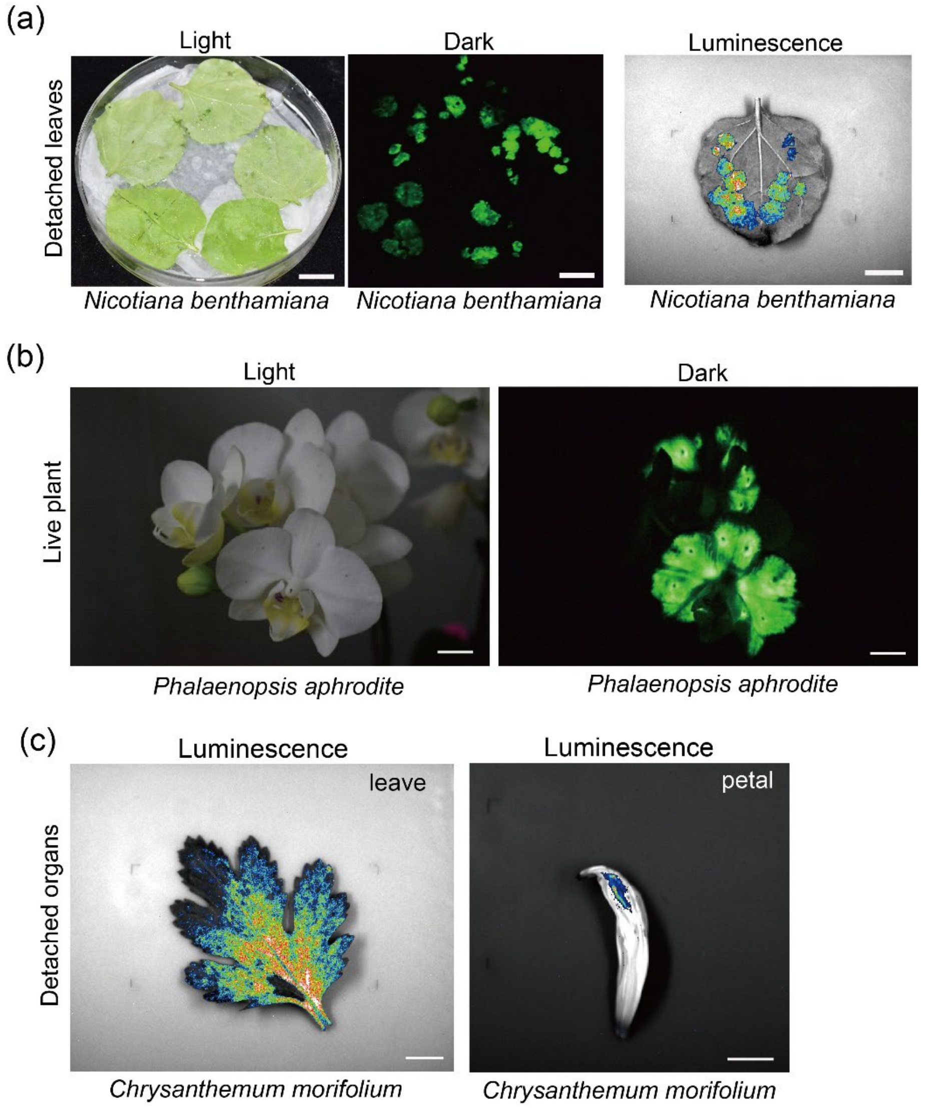

Figure S10 The test of *eFBP* module to generate luminescence in diverse plant species by transient expression.

(a-c) Bioluminescence signal image of detached leaves of *Nicotiana benthamiana* (a), flower of *Phalaenopsis Aphrodite* (b), and detached leaf and petal of *Chrysanthemum morifolium* (c) after infiltrated with *A. tumefaciens* strain EHA105 harboring the *eFBP* DNA module (Figure 1e) for 3 days. Scale bars, 1 cm.

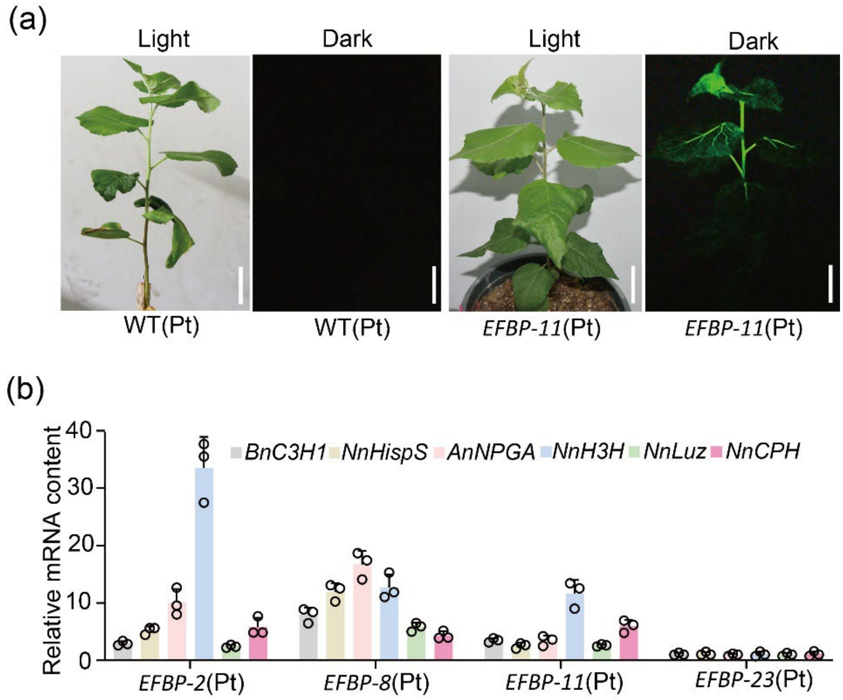

Figure S11 Identification of *eFBP* transgenic poplar lines.

(a) Appearance of wild-type and *eFBP-11* transgenic poplar sapling captured with a Nikon D750 camera with AF-S17-35mm F2.8D ED-IF at ISO 2000, F6.3 and 5-min shutter speed. Scale bars, 2 cm. Preliminary screen of 24 independent *eFBP* transgenic poplar saplings by photographic instrument.

(b) RT-qPCR assay of selected *eFBP* transgenic poplar saplings.

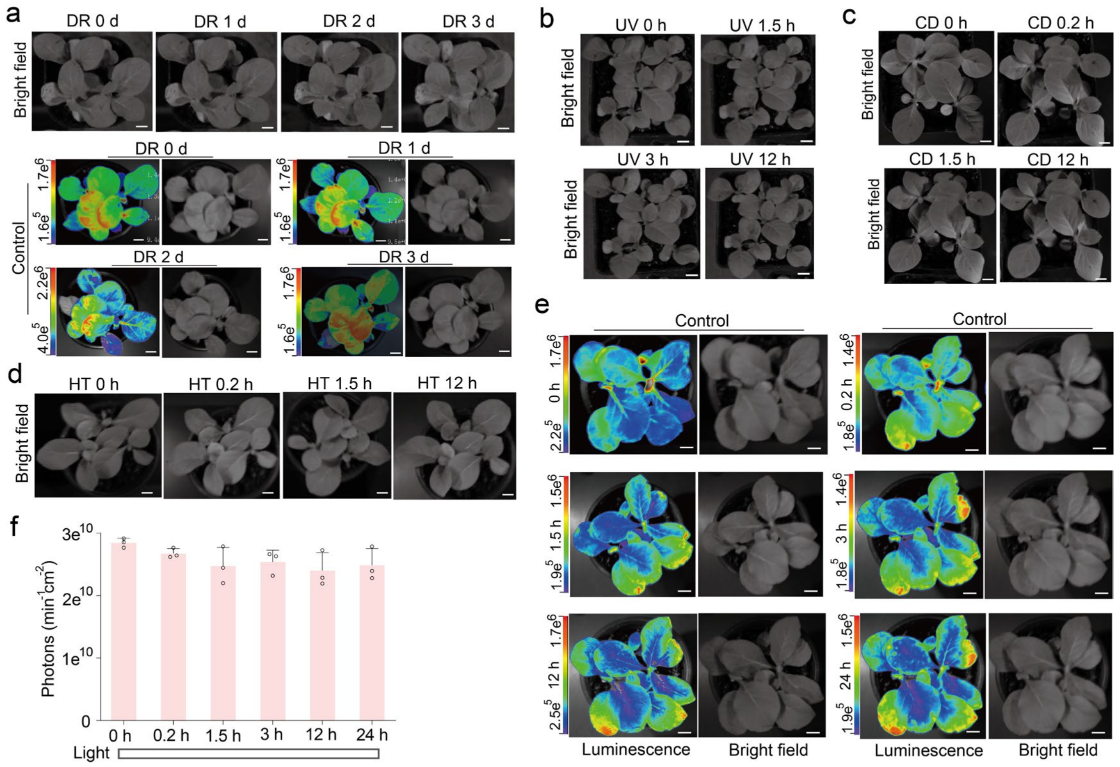

Figure S12 Analysis of the stability of *eFBP* transgenic tobacco to abiotic stresses. (a-d), The bright field of e*FBP* transgenic seedlings under diverse treatments, including drought, DR (a), ultraviolet, UV (b), cold 4 °C, CD (c), and high temperature 37 °C, HT (d). Scale bars, 1 cm. (e,f,) Bioluminescence detection of e*FBP* transgenic seedlings at normal growth conditions with different time points.

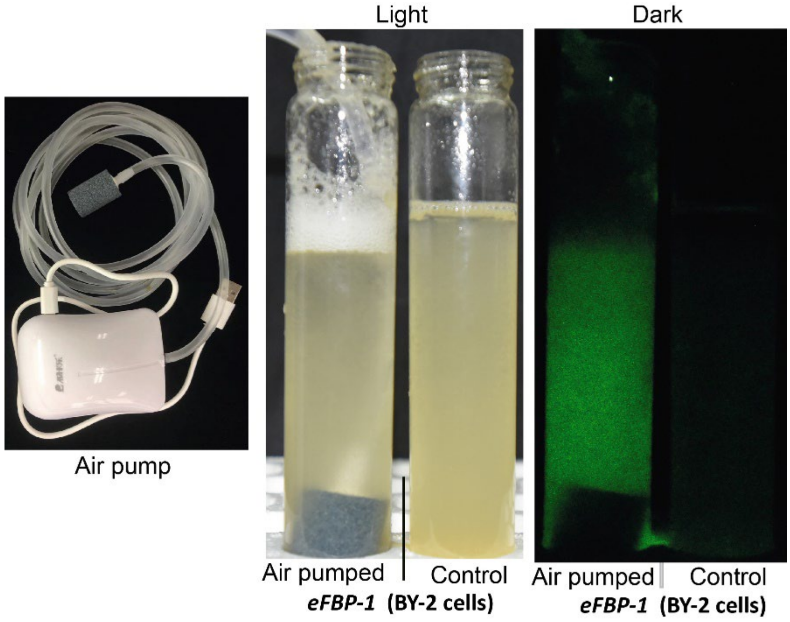

Figure S13 Oxygen requirement for bioluminescent in *eFBP* transgenic BY-2 cells. Air pump was used for adding sustained air. Control was kept quiescent for 10 sec after air

was pumped

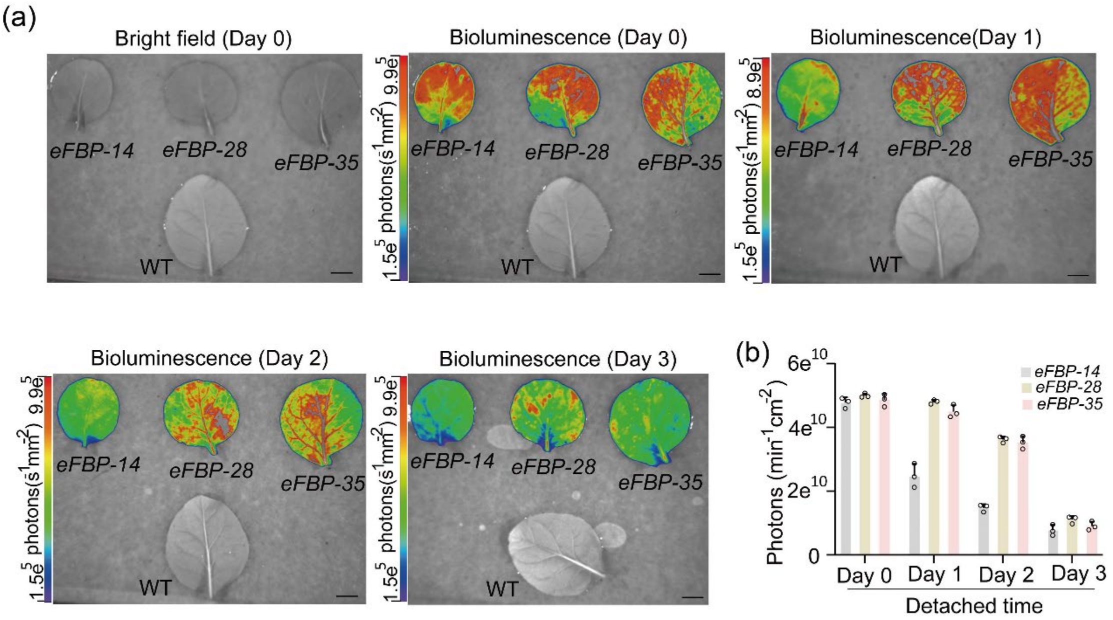

Figure S14 The stability of photon emission from detached leaves of *eFBP* transgenic tobacco seedlings.

(a) The bright field and bioluminescent images of three sizes of detached leaves from three different *eFBP* transgenic seedlings. Leaves are kept in wet and enclosed plastic boxes at room temperature with 12h light/12h dark condition.

(b) Quantitative analysis of bioluminescent intensity of detached *eFBP* transgenic leaves for three days. Scale bars, 0.5 cm. (note that the unit of colour scale indicates photon flux per sec for per square millimeter).

Video S1

The video shows immediate visualization of the auto-illumination plants in dark room. Video was taken with Sony Alpha 1, Sony GM 50mm f1.2, 1/30s f1.2 iso32000.

Supplementary Tables

Table s1 The molecular dockings of *p*-Coumaroyl shikimate into the predicted structure of C3’Hs.

The simulated docking process was performed by *AutoDock Vina* 1.2.0 with parameters "exhaustiveness=10", "num_modes=20". Only the Top-5 modes of each docking are shown here.

Protein	Mode	Affinity(kcal/mol)	dist from rmsd 1.b.	best mode rmsd u.b.
AtC3'H1	1	-8.1	0	0
	2	-8	3.382	5.611
	3	-7.7	5.426	10.408
	4	-7.4	9.491	12.188
	5	-7.4	10.294	13.008
BnC3'H1	1	-8.5	0	0
	2	-8.3	8.788	11.024
	3	-8.1	3.306	4.297
	4	-8	9.91	12.208
	5	-7.9	2.617	2.874
BdC3'H	1	-8	0	0
	2	-7.8	3.609	5.139
	3	-7.7	3.944	6.084
	4	-7.7	5.507	10.271
	5	-7.6	10.461	13.133
GmC3'H1	1	-8.4	0	0
	2	-8.3	3.066	4.921
	3	-8.2	2.275	2.563
	4	-8.1	9.76	12.622
	5	-7.9	12.811	15.372
NtC3'H1	1	-8.4	0	0
	2	-8.1	2.122	3.591
	3	-8	3.237	5.461
	4	-7.7	1.352	2.72
	5	-7.7	1.88	2.431
OsC3'H1	1	-8.2	0	0
	2	-7.8	11.651	12.885
	3	-7.8	11.442	14.144
	4	-7.8	4.471	6.4
	5	-7.8	9.459	11.404
CmC3'H	1	-8.4	0	0
	2	-8.1	8.644	11.669
	3	-8.1	2.36	8.278
	4	-7.9	4.28	8.128
	5	-7.9	3.27	7.127
TaC3'H1	1	-8	0	0
	2	-7.7	3.522	5.397

	3	-7.6	5.67	10.384
	4	-7.5	8.629	11.843
	5	-7.4	10.221	11.989
ZmC3'H1	1	-8.4	0	0
	2	-8.2	11.861	13.396
	3	-8.2	9.729	10.875
	4	-8.1	11.725	13.236
	5	-8.1	8.852	9.751
GbC3'H	1	-8	0	0
	2	-8	3.185	5.487
	3	-7.6	9.478	12.051
	4	-7.6	4.023	5.874
	5	-7.5	29.015	32.208
PpC3'H	1	-7.9	0	0
	2	-7.7	9.939	12.463
	3	-7.3	20.299	24.286
	4	-7.3	4.997	9.801
	5	-7.3	8.85	11.965
TcC3'H	1	-8.5	0	0
	2	-8.1	3.718	5.285
	3	-8	2.053	3.33
	4	-8	12.443	14.687
	5	-7.8	4.775	6.211
StC3'H1	1	-8	0	0
	2	-7.9	27.659	29.207
	3	-7.8	3.095	4.839
	4	-7.3	27.994	29.64
	5	-7.2	3.332	4.914
SlC3'H1	1	-8.4	0	0
	2	-8.2	3.227	5.41
	3	-8.2	2.151	3.677
	4	-8	12.345	15.451
	5	-8	10.886	14.385

Table S2 Vectors used in this study.

plasmid name	Fungal biolumineacent pathway components	plasmid type	Resistance
pYL380MF-oFBP	p35S:NnHisps:tNos,p35S:NnCPH:tNos,p35S:NnH3H:tNos,p35S:NnLuz:tNos	T-DNA	KmR
pYL380MF-FBP	p35S:NnHisps:tNos,p35S:NnCPH:tNos,p35S:NnH3H:tNos,p35S:AsNPGA:tNos,p35S:NnLuz:tNos	T-DNA	KmR
pYL380MF-eFBP	p35S:NnHisps:tNos,p35S:NnCPH:tNos,p35S:NnH3H:tNos,p35S:AsNPGA:tNos,p35S:NnLuz:tNos,p35S:BnC3'H1:tNos	T-DNA	KmR
d1-Hisps	p35S:NnHisps:tNos	Module	CmR
d2-CPH	p35S:NnCPH:tNos	Module	ApR
d1-H3H	p35S:NnH3H:tNos	Module	CmR
d2-NPGA	p35S:AsNPGA:tNos	Module	ApR
d1-Luz	p35S:NnLuz:tNos	Module	CmR
d2-BnC3'H1	p35S:BnC3'H1:tNos	Module	ApR

Table S3 Primers used in this study.

Primer name	Primers sequence
H3H_F	CTAGAATTCATGGCTTCATTCGAGAACTC
H3H_R	TTCGTCGACTCAAGCTGAGTTAGATGACC
HispS_F	CTAGAATTCATGAACTCATCTAAGAACCC
HispS_R	TTCGTCGACTTAGTTATCCTCTGAAGCCT
Luz_F	CTAGAATTCATGAGAATCAACATCTCACT
Luz-R	TTCGTCGACTTACTTAGCGTTCTCAACGA
NPGA_F	CAAGGATCCATGGTGCAAGATACTTCATC
NPGA_R	TTCGAATTCTCATCCCCTAGACAAACAGT
CPH_F	CAAGGATCCATGGCTCCTATCTCATCTAC
CPH_R	TTCGAATTCTTACTCATGTCCACCAGAAG
BnC3'H1_F	CAAGGATCCATGTCGTGGTTTCTTATAGC

BnC3'H1_R	TTCGAATTCTTACATTTCATAAGGCACAC
NPGA-Flag_F	CGACGGCCAGTGCCAAGCTTCATGGAGTCAAAGATTCAAATAGAGG
NPGA-Flag_R	TGGTCTTTGTAGTCGGATCCTCCCCTAGACAAACAGTTACAAAC
AtC3'H1-Flag_F	ACACGGGGGACTCCAAGGATCCATGTCGTGGTTTCTAATAGCGGTGG
AtC3'H1-Flag_R	ATGGTCTTTGTAGTCGGATCCCATATCGTAAGGCACGCGTTTGTAC
OsC3'H1-Flag_F	CACGGGGGACTCCAAGGATCCATGGACGTCGCGTCGCTGCTCCCGTT
OsC3'H1-Flag_R	ATGGTCTTTGTAGTCGGATCCCATCTCGACAGGGAACCTCTTGTAC
BnC3'H1-Flag_F	CACGGGGGACTCCAAGGATCCATGTCGTGGTTTCTTATAGCGGC
BnC3'H1-Flag_R	ATGGTCTTTGTAGTCGGATCCCATTTCATAAGGCACACGTTTAT
NtC3'H1-Flag_F	CACGGGGGACTCCAAGGATCCATGGCTATTTCTTTAGCTGCTGCA
NtC3'H1-Flag_R	ATGGTCTTTGTAGTCGGATCCCATGTCCACTGCAATTCGTTTATACA
SlC3'H1-Flag_F	CACGGGGGACTCCAAGGATCCATGGCCTTTTCTTTAGCATTTCCAGC
SlC3'H1-Flag_R	ATGGTCTTTGTAGTCGGATCCTATAACGATAGGACTGCGTTCATACAAG
SlC3'H2-Flag_F	CACGGGGGACTCCAAGGATCCATGGCTTTATTTTTAATTATTCTAACTTC
SlC3'H2-Flag_R	ATGGTCTTTGTAGTCGGATCCCATATCCATCGGCACACGTTTATACA
PAS_F	gagaaaaactagaaatttacgacatAATTTCTCCTAACTTTCACTTTGATTTTC
PAS_R	cagtaggatagaggtggctcCCATTAATCAACATACAATG
HPT_F	ACACTACATGGCGTGATTTCAT
HPT_R	TCCACTATCGGCGAGTACTTCT
SLP8rt_F	GGGTTGAATTTGGGCGGAAG
SLP8rt_R	TCGGTGGTAGTGCTTTGCAT
NtHCTrt_F	GCCTATTTGGTATGCTGCCAGT
NtHCTrt_R	GCATCATGGATTGGCAGCCT
NtCSErt_F	CAGGAGATATACCGGGAAGCC
NtCSErt_R	TGTAGGGCATGTAACACCATCAGA
NtC4Hrt_F	CCAGGAGTGCAAGTGACTGAA
NtC4Hrt_R	TGCTGGAATATCAAACCCGCC
NtPATrt_F	CGTGAGCGACGTGATTACCTT
NtPATrt_R	TCTCCTGGGACAAGCGCTAC

NtADT1rt_F	GTGCTCGAGCTGCAGAGATT
NtADT1rt_R	TCCAGGCCCTTCTTCCAGAG
NtADT2rt_F	AAACTAAAAGATGCAGGGGCAGT
NtADT2rt_R	CCTTCATCGAGCGAAAAGACCA
NtCM1rt_F	GGCTGCCATAAGAGCACAAGAC
NtCM1rt_R	GTCGCCGTACAATTCAGCAACTA
NtCM2rt_F	GAGTTGCAAAGAAAGCCTTGACG
NtCM2rt_R	GCGCGAAACTAGCGATGGAT
NtPAL1rt_F	ACGCTGATGATCCTTGCAGT
NtPAL1rt_R	ACTTTCTAATGCAGCTCTTGCAC
NtPAL2rt_F	GACAAGTGTTGGTGGATCATGC
NtPAL2rt_R	CCAAGTCACATCTAGCACTCTCA
NtC3'H1rt_F	CCATTGGAGTTCAGACCGGAG
NtC3'H1rt_R	AAATGGTGCAAAAGATGGCCC
NtC3'H2rt_F	GCCGGAAGACGTATATGTCCAG
NtC3'H2rt_R	GCATGTAGGTGACTGTACCAGGA
NtC3'H3rt_F	TGGTAGGCGTATTTGTCCTGGTAC
NtC3'H3rt_R	AGTAGGCACAGCTTGTAGAGGC
NtC3'H4rt_F	CAGGAAGGCGTGTTTGCCC
NtC3'H4rt_R	TGTTCCAGGGCTCTCCTCC
EF1art_F	CTGCAACAAGATGGATGCTAC
EF1art_R	ACCAACAGGGACAGTACCAAT
BnC3'H1rt_F	CGGGTATGGACACAACAGCT
BnC3'H1rt_R	GTGCGTTGGATCTGTGAGGA
Luzrt_F	TCCTCCTCCCTTCAATCCCA
Luzrt_R	AGCCTCCTCCTCGTTCCTAG
NPGArt_F	TCAGGGAGCTGGAGTTCTCA
NPGArt_R	CTGGCCTAGATGAAGCTCCG
H3Hrt_F	TGATTGGTCAGTGCCAGCTT

H3Hrt_R	CGAGAGCCACAGCATCTTCA
CPHrt_F	GTTGAACGGTGGAGTTGTGC
CPHrt_R	ACTCATGTCCACCAGAAGCG
HISPSrt_F	TGTGCAATTCGCTCAAGCTG
HISPSrt_R	TGCATCCTCTTGTTGAGCGT
PtACTINrt_F	AAACTGTAATGGTCCTCCCTCCG
PtACTINrt_R	GCATCATCACAATCACTCTCCGA
HispS-YFP_F	GAGAACACGGGGGACTCTAGAATGAACTCATCTAAGAACCCACCAT
HispS-YFP_R	TTGCTCACCATTGTTGGATCGTTATCCTCTGAAGCCTCAGCTTG
