# Supplementary figures and images for: Metabolic engineering and mechanical investigation of enhanced plant autoluminescence
Source: Plant Biotechnol J. 2023 May 8;21(8):1671–81. doi: 10.1111/pbi.14068 (PMC10363767; doi:10.1111/pbi.14068)

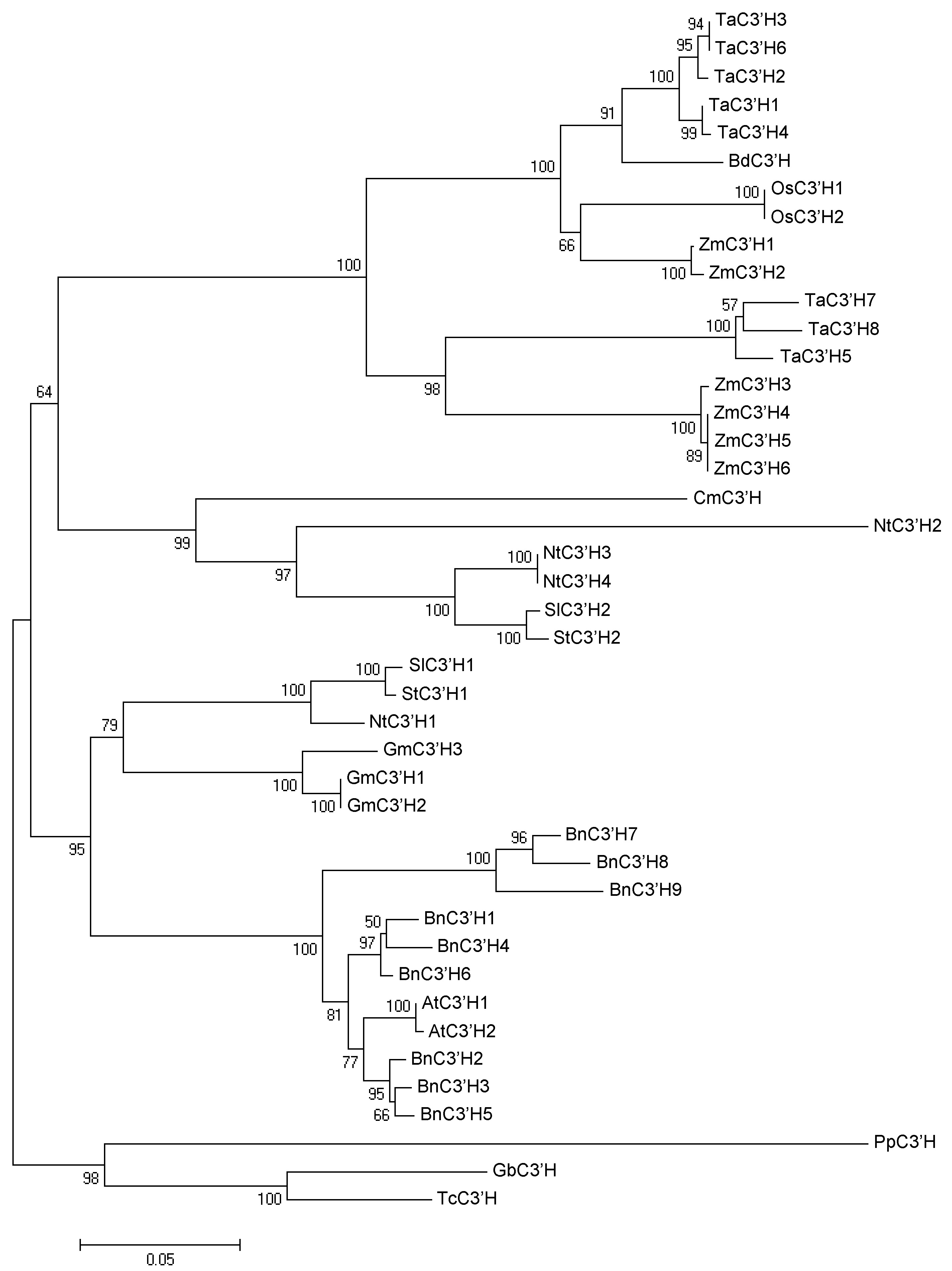

Supplement: Supplementary file 1 — Figure S1 Protein sequences cluster of C3′H homologues. Figure S2 Transiently expressing C3′H1 constructs for enzyme activity assay. Figure S3 Molecular modelling of BnC3′H1. Figure S4 Multiple sequence alignment of C3′H homologues. Figure S5 Identification of the FBP and eFBP DNA modules and transgenic tobacco lines. Figure S6 Identification of the FBP and eFBP transgenic tobacco lines. Figure S7 FBP and eFBP transgenic lines at the flowering stage. Figure S8 Characterization of selectable marker excised plants from eFBP transgenic lines. Figure S9 Analysis of the light emission from FBP and eFBP BY‐2 cell lines. Figure S10 The test of eFBP module to generate luminescence in diverse plant species by transient expression. Figure S11 Identification of eFBP transgenic poplar lines. Figure S12 Analysis of the stability of eFBP transgenic tobacco to abiotic stresses. Figure S13 Oxygen requirement for bioluminescent in eFBP transgenic BY‐2 cells. Figure S14 The stability of photon emission from detached leaves of eFBP transgenic tobacco seedlings. Video S1 The video shows immediate visualization of the auto‐illumination plants in dark room. Table S1 The molecular dockings of p‐Coumaroyl shikimate into the predicted structure of C3'Hs. Table S2 Vectors used in this study. Table S3 Primers used in this study. [file PBI-21-1671-s001.zip › Figure S1.jpg]

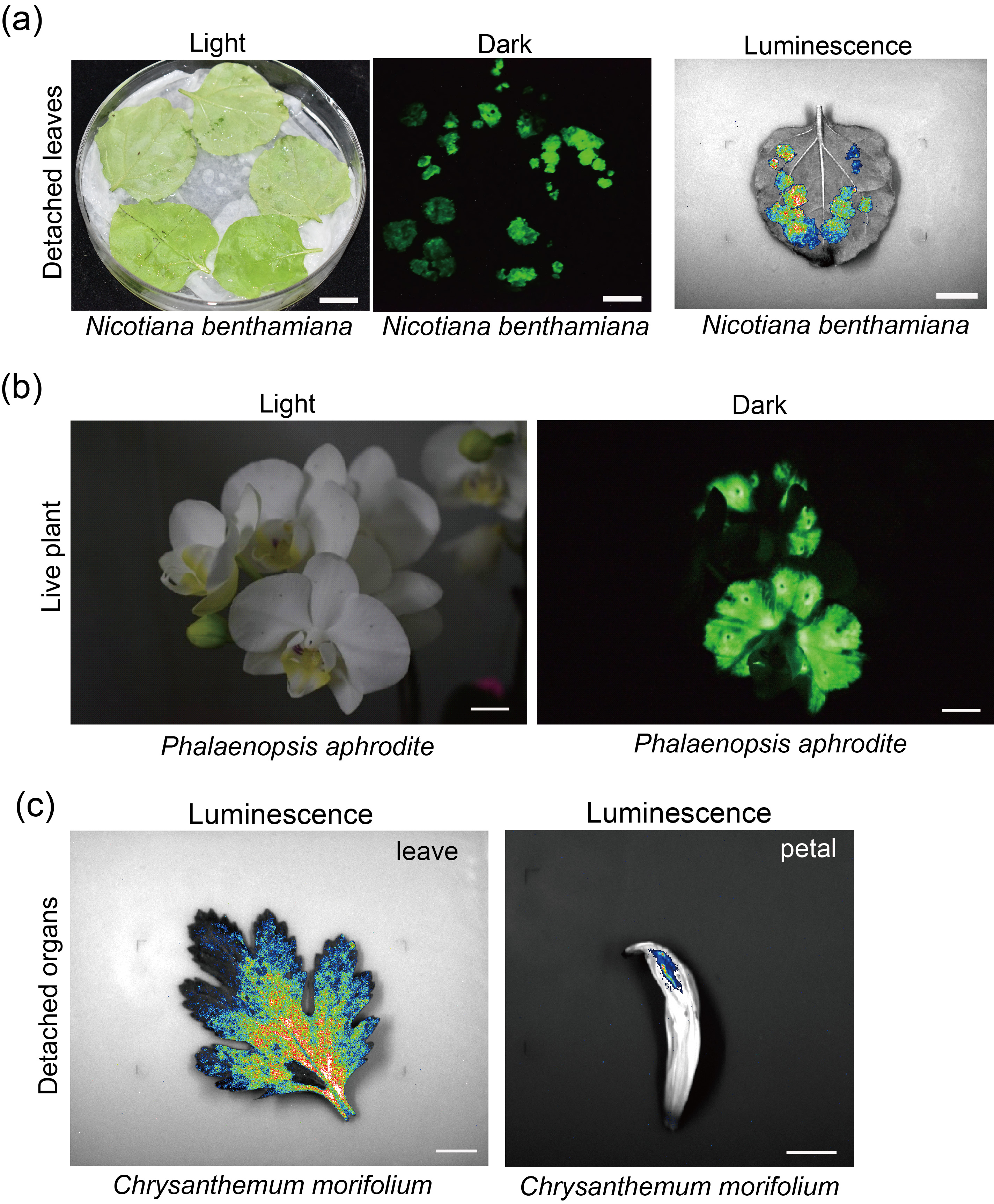

Supplement: Supplementary file 1 — Figure S1 Protein sequences cluster of C3′H homologues. Figure S2 Transiently expressing C3′H1 constructs for enzyme activity assay. Figure S3 Molecular modelling of BnC3′H1. Figure S4 Multiple sequence alignment of C3′H homologues. Figure S5 Identification of the FBP and eFBP DNA modules and transgenic tobacco lines. Figure S6 Identification of the FBP and eFBP transgenic tobacco lines. Figure S7 FBP and eFBP transgenic lines at the flowering stage. Figure S8 Characterization of selectable marker excised plants from eFBP transgenic lines. Figure S9 Analysis of the light emission from FBP and eFBP BY‐2 cell lines. Figure S10 The test of eFBP module to generate luminescence in diverse plant species by transient expression. Figure S11 Identification of eFBP transgenic poplar lines. Figure S12 Analysis of the stability of eFBP transgenic tobacco to abiotic stresses. Figure S13 Oxygen requirement for bioluminescent in eFBP transgenic BY‐2 cells. Figure S14 The stability of photon emission from detached leaves of eFBP transgenic tobacco seedlings. Video S1 The video shows immediate visualization of the auto‐illumination plants in dark room. Table S1 The molecular dockings of p‐Coumaroyl shikimate into the predicted structure of C3'Hs. Table S2 Vectors used in this study. Table S3 Primers used in this study. [file PBI-21-1671-s001.zip › Figure S10.jpg]

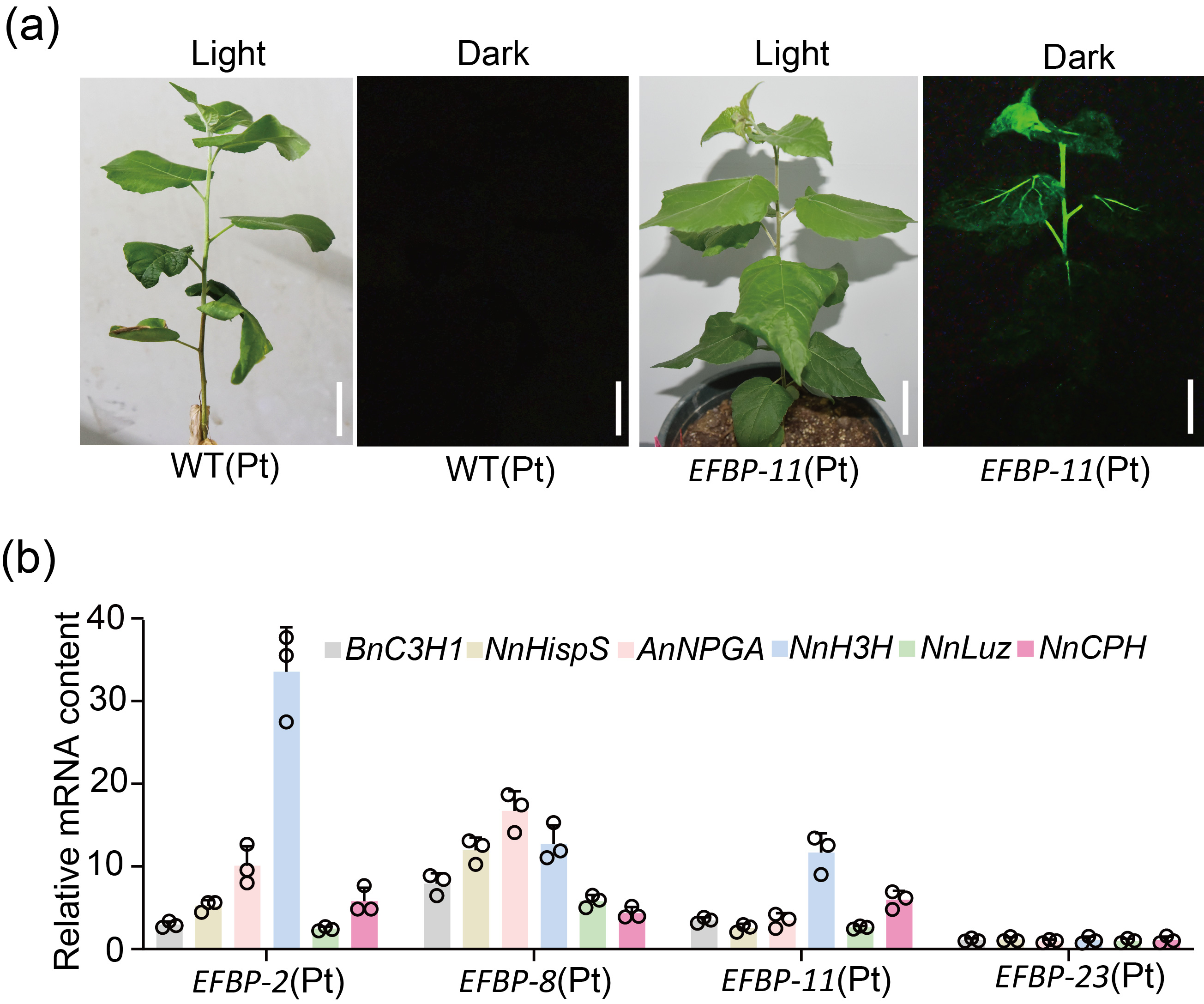

Supplement: Supplementary file 1 — Figure S1 Protein sequences cluster of C3′H homologues. Figure S2 Transiently expressing C3′H1 constructs for enzyme activity assay. Figure S3 Molecular modelling of BnC3′H1. Figure S4 Multiple sequence alignment of C3′H homologues. Figure S5 Identification of the FBP and eFBP DNA modules and transgenic tobacco lines. Figure S6 Identification of the FBP and eFBP transgenic tobacco lines. Figure S7 FBP and eFBP transgenic lines at the flowering stage. Figure S8 Characterization of selectable marker excised plants from eFBP transgenic lines. Figure S9 Analysis of the light emission from FBP and eFBP BY‐2 cell lines. Figure S10 The test of eFBP module to generate luminescence in diverse plant species by transient expression. Figure S11 Identification of eFBP transgenic poplar lines. Figure S12 Analysis of the stability of eFBP transgenic tobacco to abiotic stresses. Figure S13 Oxygen requirement for bioluminescent in eFBP transgenic BY‐2 cells. Figure S14 The stability of photon emission from detached leaves of eFBP transgenic tobacco seedlings. Video S1 The video shows immediate visualization of the auto‐illumination plants in dark room. Table S1 The molecular dockings of p‐Coumaroyl shikimate into the predicted structure of C3'Hs. Table S2 Vectors used in this study. Table S3 Primers used in this study. [file PBI-21-1671-s001.zip › Figure S11.jpg]

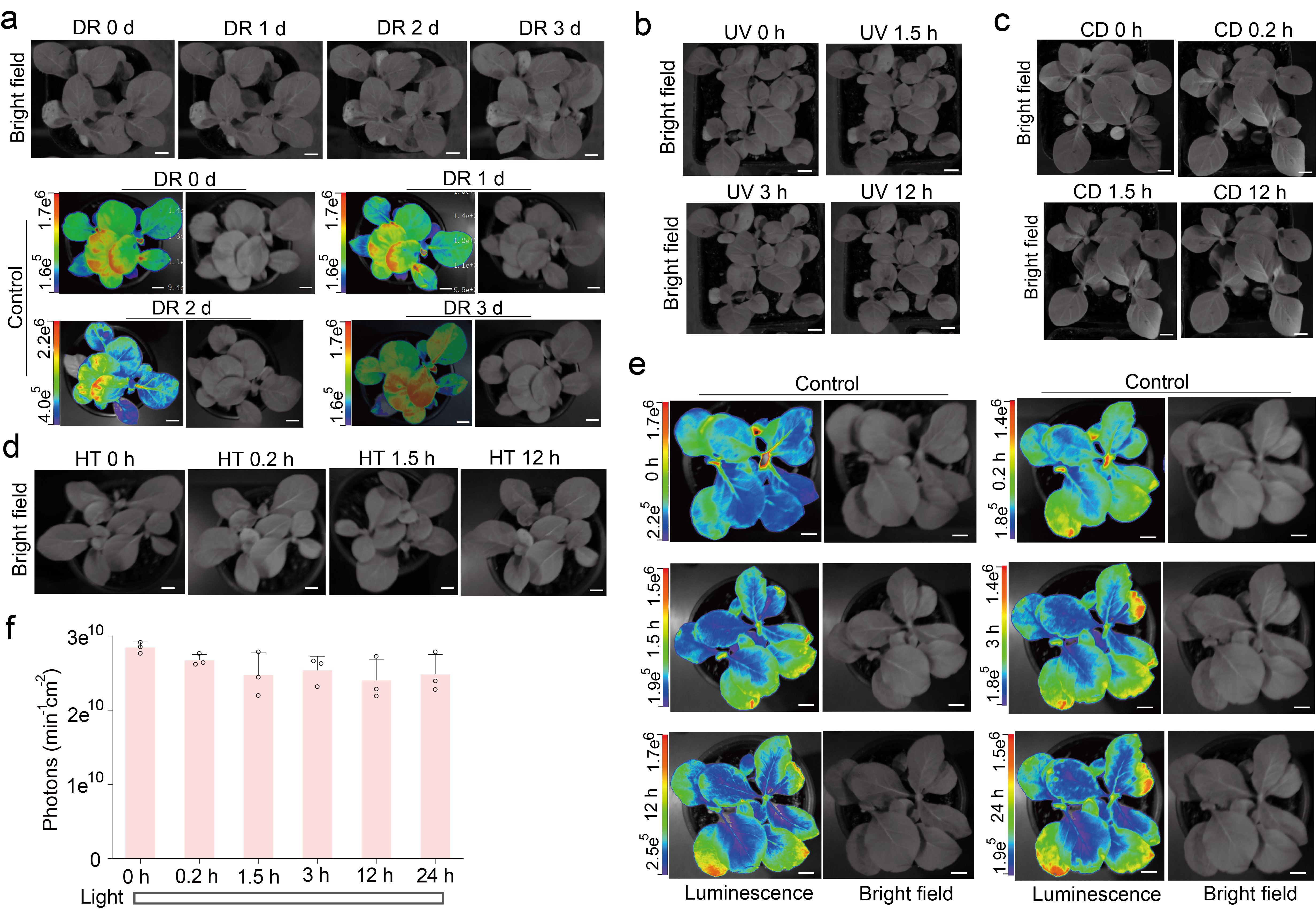

Supplement: Supplementary file 1 — Figure S1 Protein sequences cluster of C3′H homologues. Figure S2 Transiently expressing C3′H1 constructs for enzyme activity assay. Figure S3 Molecular modelling of BnC3′H1. Figure S4 Multiple sequence alignment of C3′H homologues. Figure S5 Identification of the FBP and eFBP DNA modules and transgenic tobacco lines. Figure S6 Identification of the FBP and eFBP transgenic tobacco lines. Figure S7 FBP and eFBP transgenic lines at the flowering stage. Figure S8 Characterization of selectable marker excised plants from eFBP transgenic lines. Figure S9 Analysis of the light emission from FBP and eFBP BY‐2 cell lines. Figure S10 The test of eFBP module to generate luminescence in diverse plant species by transient expression. Figure S11 Identification of eFBP transgenic poplar lines. Figure S12 Analysis of the stability of eFBP transgenic tobacco to abiotic stresses. Figure S13 Oxygen requirement for bioluminescent in eFBP transgenic BY‐2 cells. Figure S14 The stability of photon emission from detached leaves of eFBP transgenic tobacco seedlings. Video S1 The video shows immediate visualization of the auto‐illumination plants in dark room. Table S1 The molecular dockings of p‐Coumaroyl shikimate into the predicted structure of C3'Hs. Table S2 Vectors used in this study. Table S3 Primers used in this study. [file PBI-21-1671-s001.zip › Figure S12.jpg]

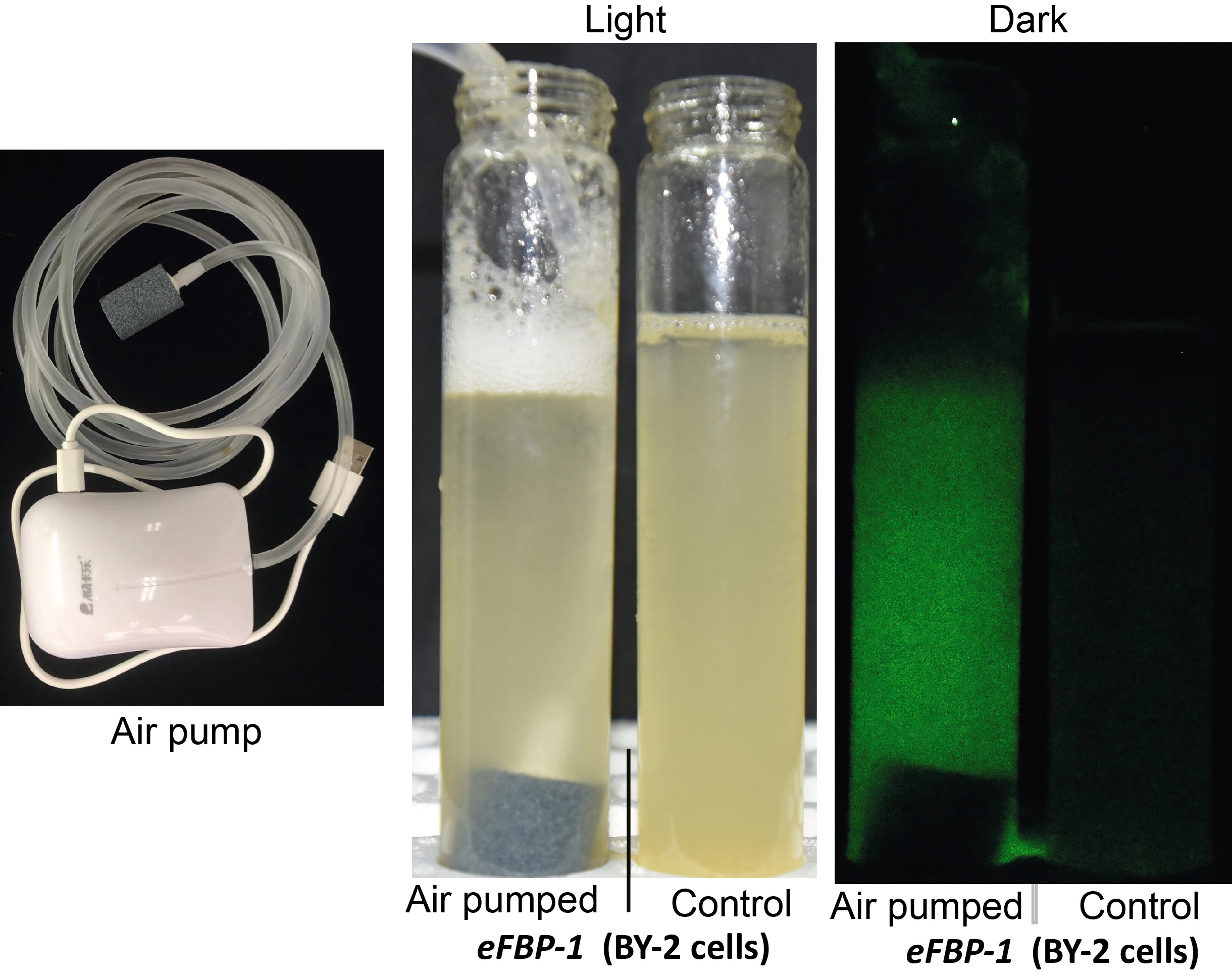

Supplement: Supplementary file 1 — Figure S1 Protein sequences cluster of C3′H homologues. Figure S2 Transiently expressing C3′H1 constructs for enzyme activity assay. Figure S3 Molecular modelling of BnC3′H1. Figure S4 Multiple sequence alignment of C3′H homologues. Figure S5 Identification of the FBP and eFBP DNA modules and transgenic tobacco lines. Figure S6 Identification of the FBP and eFBP transgenic tobacco lines. Figure S7 FBP and eFBP transgenic lines at the flowering stage. Figure S8 Characterization of selectable marker excised plants from eFBP transgenic lines. Figure S9 Analysis of the light emission from FBP and eFBP BY‐2 cell lines. Figure S10 The test of eFBP module to generate luminescence in diverse plant species by transient expression. Figure S11 Identification of eFBP transgenic poplar lines. Figure S12 Analysis of the stability of eFBP transgenic tobacco to abiotic stresses. Figure S13 Oxygen requirement for bioluminescent in eFBP transgenic BY‐2 cells. Figure S14 The stability of photon emission from detached leaves of eFBP transgenic tobacco seedlings. Video S1 The video shows immediate visualization of the auto‐illumination plants in dark room. Table S1 The molecular dockings of p‐Coumaroyl shikimate into the predicted structure of C3'Hs. Table S2 Vectors used in this study. Table S3 Primers used in this study. [file PBI-21-1671-s001.zip › Figure S13.jpg]

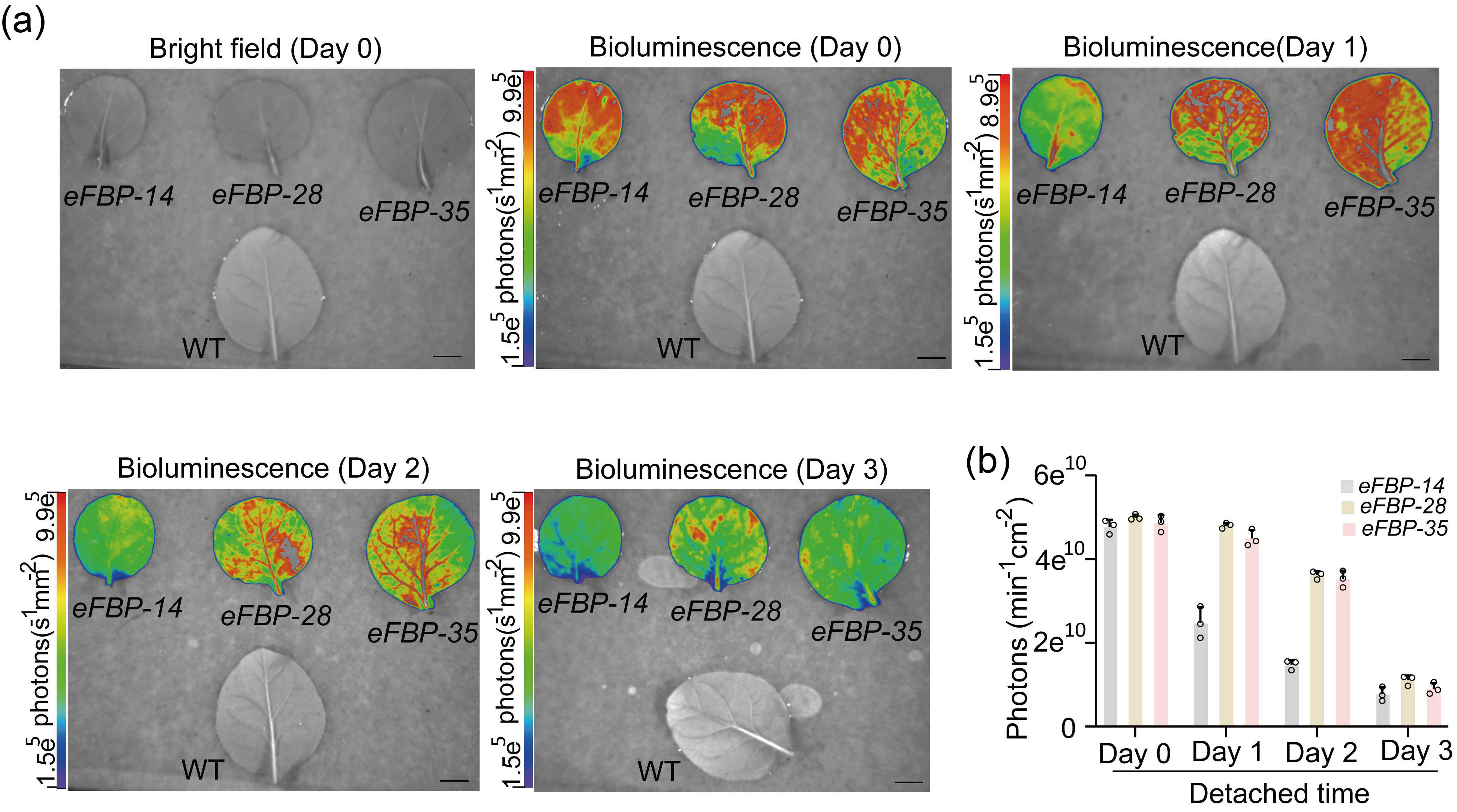

Supplement: Supplementary file 1 — Figure S1 Protein sequences cluster of C3′H homologues. Figure S2 Transiently expressing C3′H1 constructs for enzyme activity assay. Figure S3 Molecular modelling of BnC3′H1. Figure S4 Multiple sequence alignment of C3′H homologues. Figure S5 Identification of the FBP and eFBP DNA modules and transgenic tobacco lines. Figure S6 Identification of the FBP and eFBP transgenic tobacco lines. Figure S7 FBP and eFBP transgenic lines at the flowering stage. Figure S8 Characterization of selectable marker excised plants from eFBP transgenic lines. Figure S9 Analysis of the light emission from FBP and eFBP BY‐2 cell lines. Figure S10 The test of eFBP module to generate luminescence in diverse plant species by transient expression. Figure S11 Identification of eFBP transgenic poplar lines. Figure S12 Analysis of the stability of eFBP transgenic tobacco to abiotic stresses. Figure S13 Oxygen requirement for bioluminescent in eFBP transgenic BY‐2 cells. Figure S14 The stability of photon emission from detached leaves of eFBP transgenic tobacco seedlings. Video S1 The video shows immediate visualization of the auto‐illumination plants in dark room. Table S1 The molecular dockings of p‐Coumaroyl shikimate into the predicted structure of C3'Hs. Table S2 Vectors used in this study. Table S3 Primers used in this study. [file PBI-21-1671-s001.zip › Figure S14.jpg]

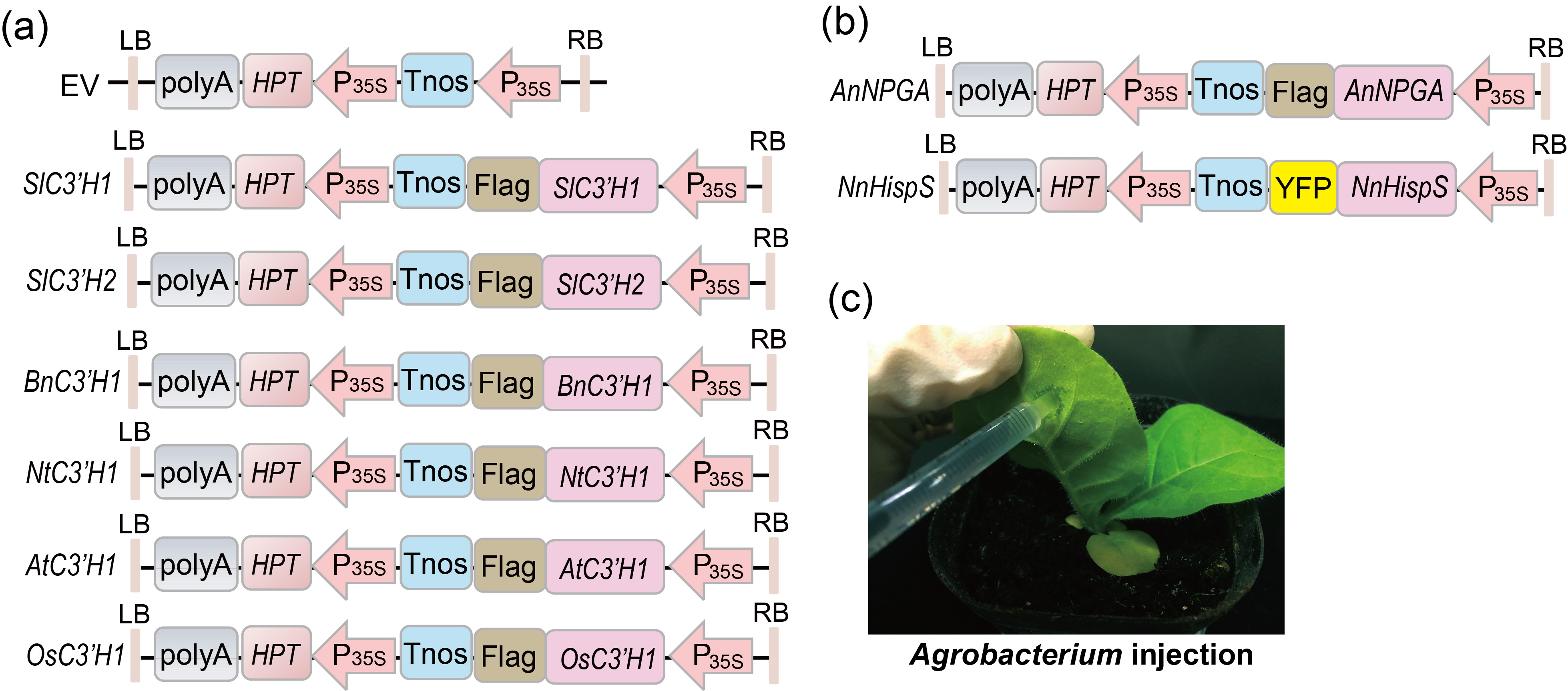

Supplement: Supplementary file 1 — Figure S1 Protein sequences cluster of C3′H homologues. Figure S2 Transiently expressing C3′H1 constructs for enzyme activity assay. Figure S3 Molecular modelling of BnC3′H1. Figure S4 Multiple sequence alignment of C3′H homologues. Figure S5 Identification of the FBP and eFBP DNA modules and transgenic tobacco lines. Figure S6 Identification of the FBP and eFBP transgenic tobacco lines. Figure S7 FBP and eFBP transgenic lines at the flowering stage. Figure S8 Characterization of selectable marker excised plants from eFBP transgenic lines. Figure S9 Analysis of the light emission from FBP and eFBP BY‐2 cell lines. Figure S10 The test of eFBP module to generate luminescence in diverse plant species by transient expression. Figure S11 Identification of eFBP transgenic poplar lines. Figure S12 Analysis of the stability of eFBP transgenic tobacco to abiotic stresses. Figure S13 Oxygen requirement for bioluminescent in eFBP transgenic BY‐2 cells. Figure S14 The stability of photon emission from detached leaves of eFBP transgenic tobacco seedlings. Video S1 The video shows immediate visualization of the auto‐illumination plants in dark room. Table S1 The molecular dockings of p‐Coumaroyl shikimate into the predicted structure of C3'Hs. Table S2 Vectors used in this study. Table S3 Primers used in this study. [file PBI-21-1671-s001.zip › Figure S2.jpg]

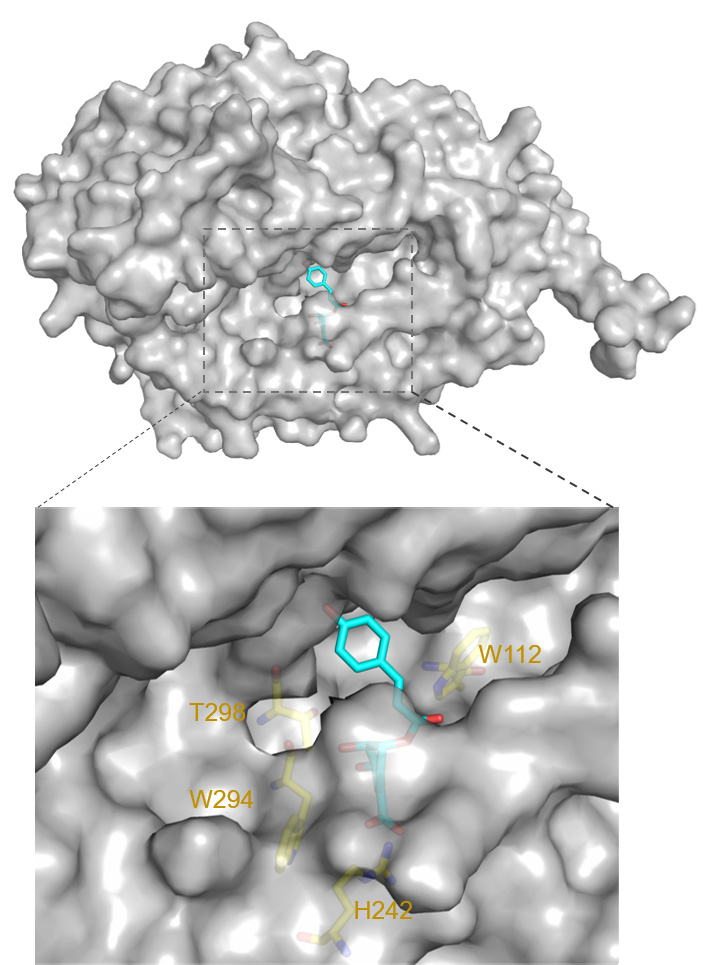

Supplement: Supplementary file 1 — Figure S1 Protein sequences cluster of C3′H homologues. Figure S2 Transiently expressing C3′H1 constructs for enzyme activity assay. Figure S3 Molecular modelling of BnC3′H1. Figure S4 Multiple sequence alignment of C3′H homologues. Figure S5 Identification of the FBP and eFBP DNA modules and transgenic tobacco lines. Figure S6 Identification of the FBP and eFBP transgenic tobacco lines. Figure S7 FBP and eFBP transgenic lines at the flowering stage. Figure S8 Characterization of selectable marker excised plants from eFBP transgenic lines. Figure S9 Analysis of the light emission from FBP and eFBP BY‐2 cell lines. Figure S10 The test of eFBP module to generate luminescence in diverse plant species by transient expression. Figure S11 Identification of eFBP transgenic poplar lines. Figure S12 Analysis of the stability of eFBP transgenic tobacco to abiotic stresses. Figure S13 Oxygen requirement for bioluminescent in eFBP transgenic BY‐2 cells. Figure S14 The stability of photon emission from detached leaves of eFBP transgenic tobacco seedlings. Video S1 The video shows immediate visualization of the auto‐illumination plants in dark room. Table S1 The molecular dockings of p‐Coumaroyl shikimate into the predicted structure of C3'Hs. Table S2 Vectors used in this study. Table S3 Primers used in this study. [file PBI-21-1671-s001.zip › Figure S3.jpg]

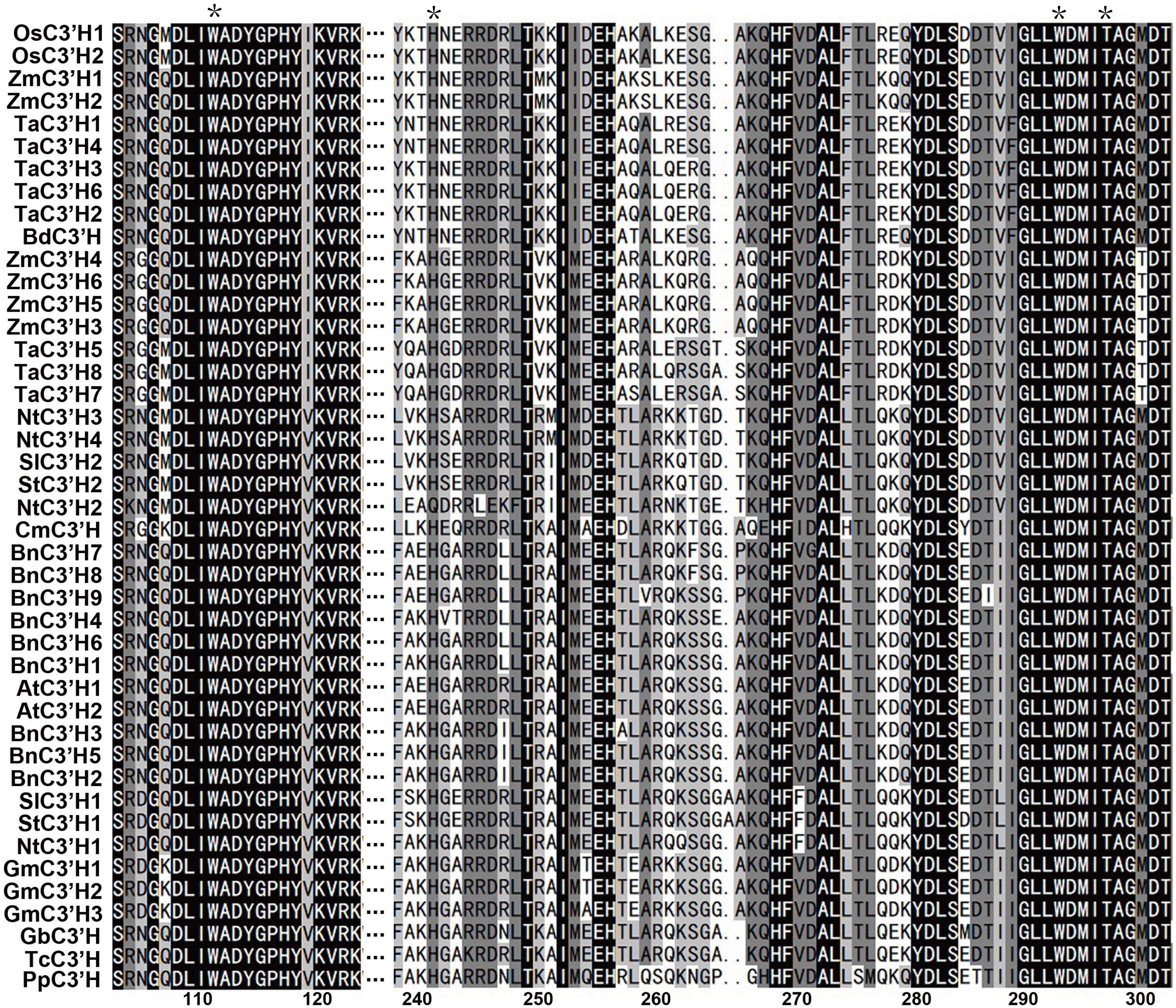

Supplement: Supplementary file 1 — Figure S1 Protein sequences cluster of C3′H homologues. Figure S2 Transiently expressing C3′H1 constructs for enzyme activity assay. Figure S3 Molecular modelling of BnC3′H1. Figure S4 Multiple sequence alignment of C3′H homologues. Figure S5 Identification of the FBP and eFBP DNA modules and transgenic tobacco lines. Figure S6 Identification of the FBP and eFBP transgenic tobacco lines. Figure S7 FBP and eFBP transgenic lines at the flowering stage. Figure S8 Characterization of selectable marker excised plants from eFBP transgenic lines. Figure S9 Analysis of the light emission from FBP and eFBP BY‐2 cell lines. Figure S10 The test of eFBP module to generate luminescence in diverse plant species by transient expression. Figure S11 Identification of eFBP transgenic poplar lines. Figure S12 Analysis of the stability of eFBP transgenic tobacco to abiotic stresses. Figure S13 Oxygen requirement for bioluminescent in eFBP transgenic BY‐2 cells. Figure S14 The stability of photon emission from detached leaves of eFBP transgenic tobacco seedlings. Video S1 The video shows immediate visualization of the auto‐illumination plants in dark room. Table S1 The molecular dockings of p‐Coumaroyl shikimate into the predicted structure of C3'Hs. Table S2 Vectors used in this study. Table S3 Primers used in this study. [file PBI-21-1671-s001.zip › Figure S4.jpg]

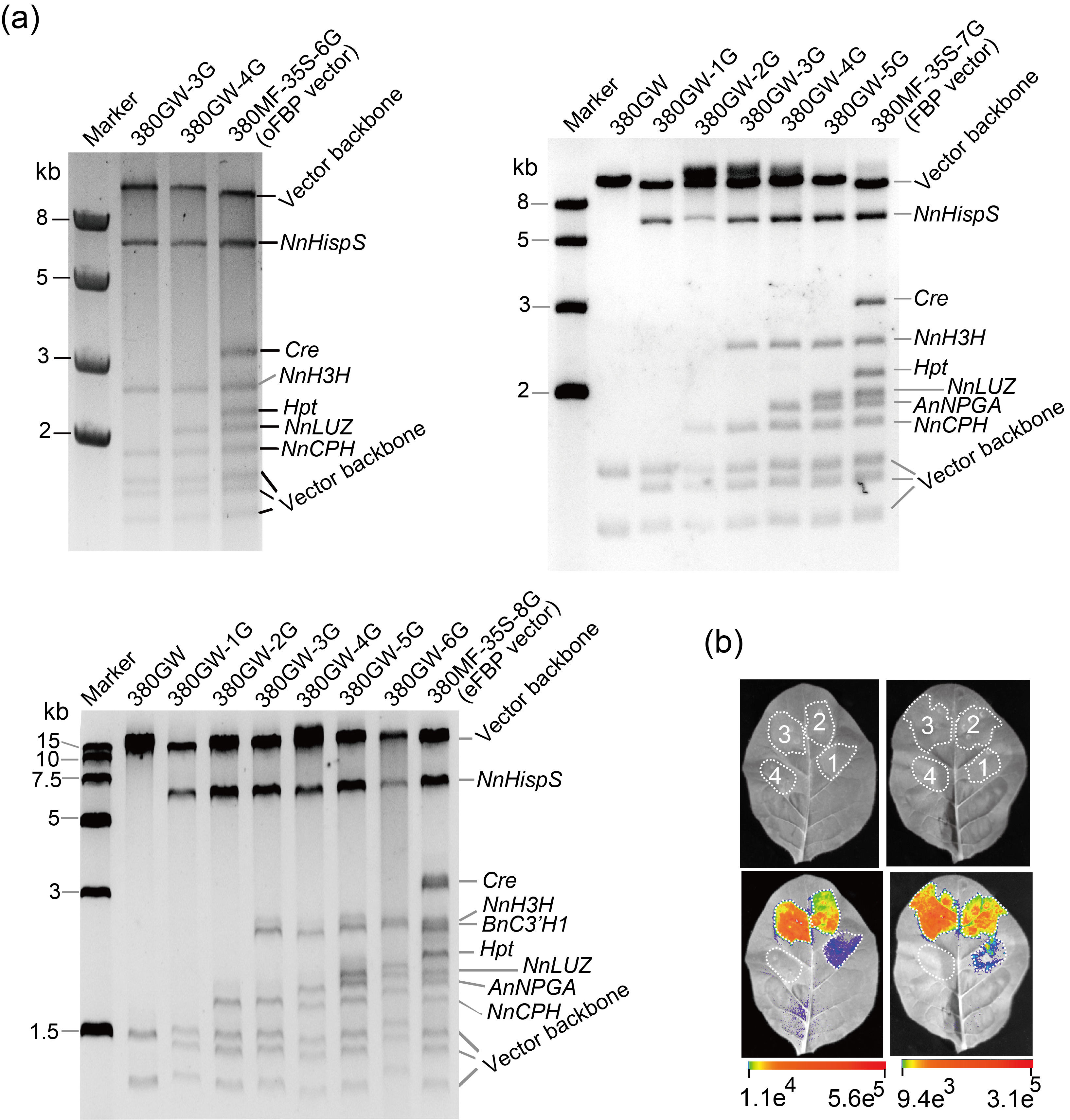

Supplement: Supplementary file 1 — Figure S1 Protein sequences cluster of C3′H homologues. Figure S2 Transiently expressing C3′H1 constructs for enzyme activity assay. Figure S3 Molecular modelling of BnC3′H1. Figure S4 Multiple sequence alignment of C3′H homologues. Figure S5 Identification of the FBP and eFBP DNA modules and transgenic tobacco lines. Figure S6 Identification of the FBP and eFBP transgenic tobacco lines. Figure S7 FBP and eFBP transgenic lines at the flowering stage. Figure S8 Characterization of selectable marker excised plants from eFBP transgenic lines. Figure S9 Analysis of the light emission from FBP and eFBP BY‐2 cell lines. Figure S10 The test of eFBP module to generate luminescence in diverse plant species by transient expression. Figure S11 Identification of eFBP transgenic poplar lines. Figure S12 Analysis of the stability of eFBP transgenic tobacco to abiotic stresses. Figure S13 Oxygen requirement for bioluminescent in eFBP transgenic BY‐2 cells. Figure S14 The stability of photon emission from detached leaves of eFBP transgenic tobacco seedlings. Video S1 The video shows immediate visualization of the auto‐illumination plants in dark room. Table S1 The molecular dockings of p‐Coumaroyl shikimate into the predicted structure of C3'Hs. Table S2 Vectors used in this study. Table S3 Primers used in this study. [file PBI-21-1671-s001.zip › Figure S5.jpg]

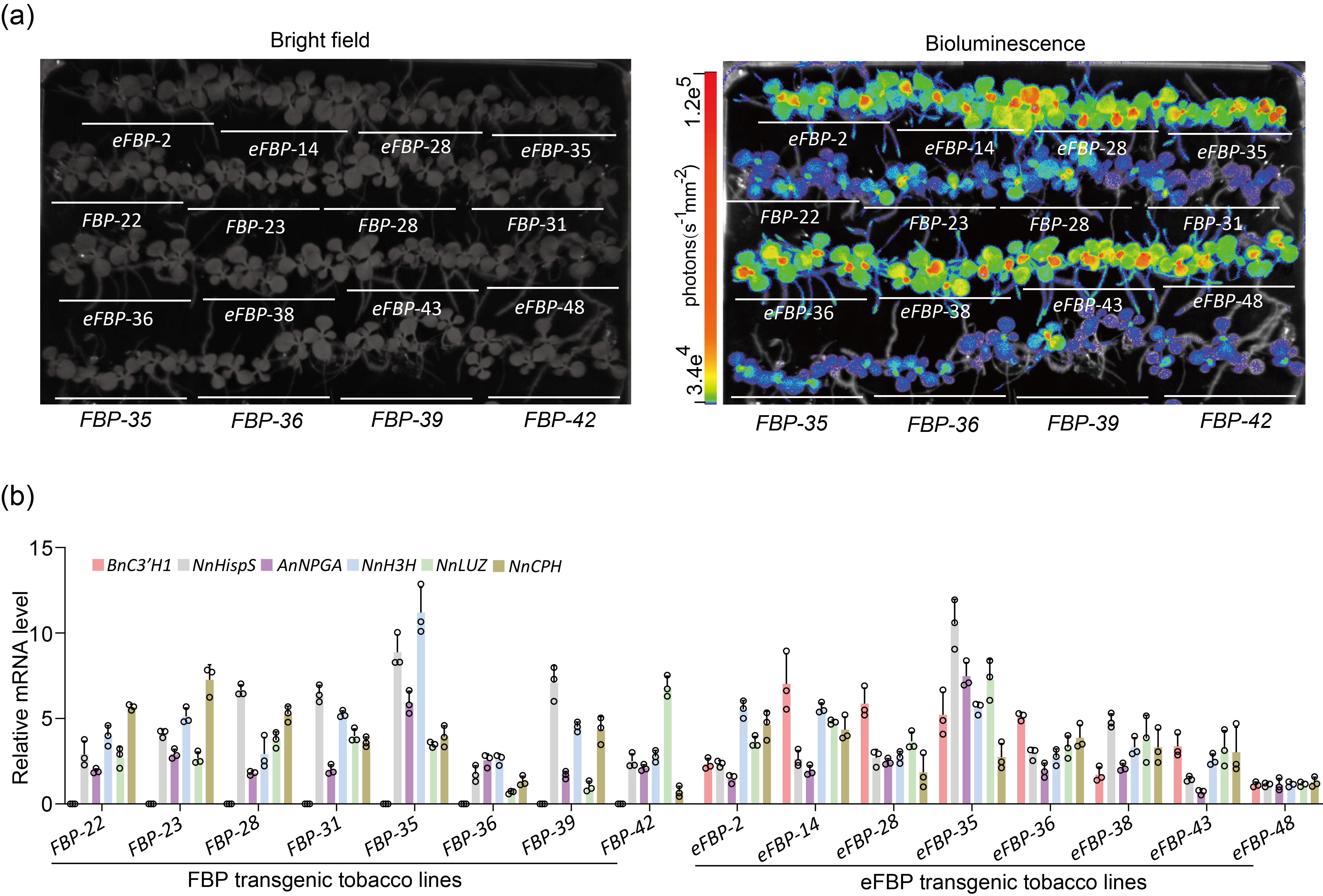

Supplement: Supplementary file 1 — Figure S1 Protein sequences cluster of C3′H homologues. Figure S2 Transiently expressing C3′H1 constructs for enzyme activity assay. Figure S3 Molecular modelling of BnC3′H1. Figure S4 Multiple sequence alignment of C3′H homologues. Figure S5 Identification of the FBP and eFBP DNA modules and transgenic tobacco lines. Figure S6 Identification of the FBP and eFBP transgenic tobacco lines. Figure S7 FBP and eFBP transgenic lines at the flowering stage. Figure S8 Characterization of selectable marker excised plants from eFBP transgenic lines. Figure S9 Analysis of the light emission from FBP and eFBP BY‐2 cell lines. Figure S10 The test of eFBP module to generate luminescence in diverse plant species by transient expression. Figure S11 Identification of eFBP transgenic poplar lines. Figure S12 Analysis of the stability of eFBP transgenic tobacco to abiotic stresses. Figure S13 Oxygen requirement for bioluminescent in eFBP transgenic BY‐2 cells. Figure S14 The stability of photon emission from detached leaves of eFBP transgenic tobacco seedlings. Video S1 The video shows immediate visualization of the auto‐illumination plants in dark room. Table S1 The molecular dockings of p‐Coumaroyl shikimate into the predicted structure of C3'Hs. Table S2 Vectors used in this study. Table S3 Primers used in this study. [file PBI-21-1671-s001.zip › Figure S6.jpg]

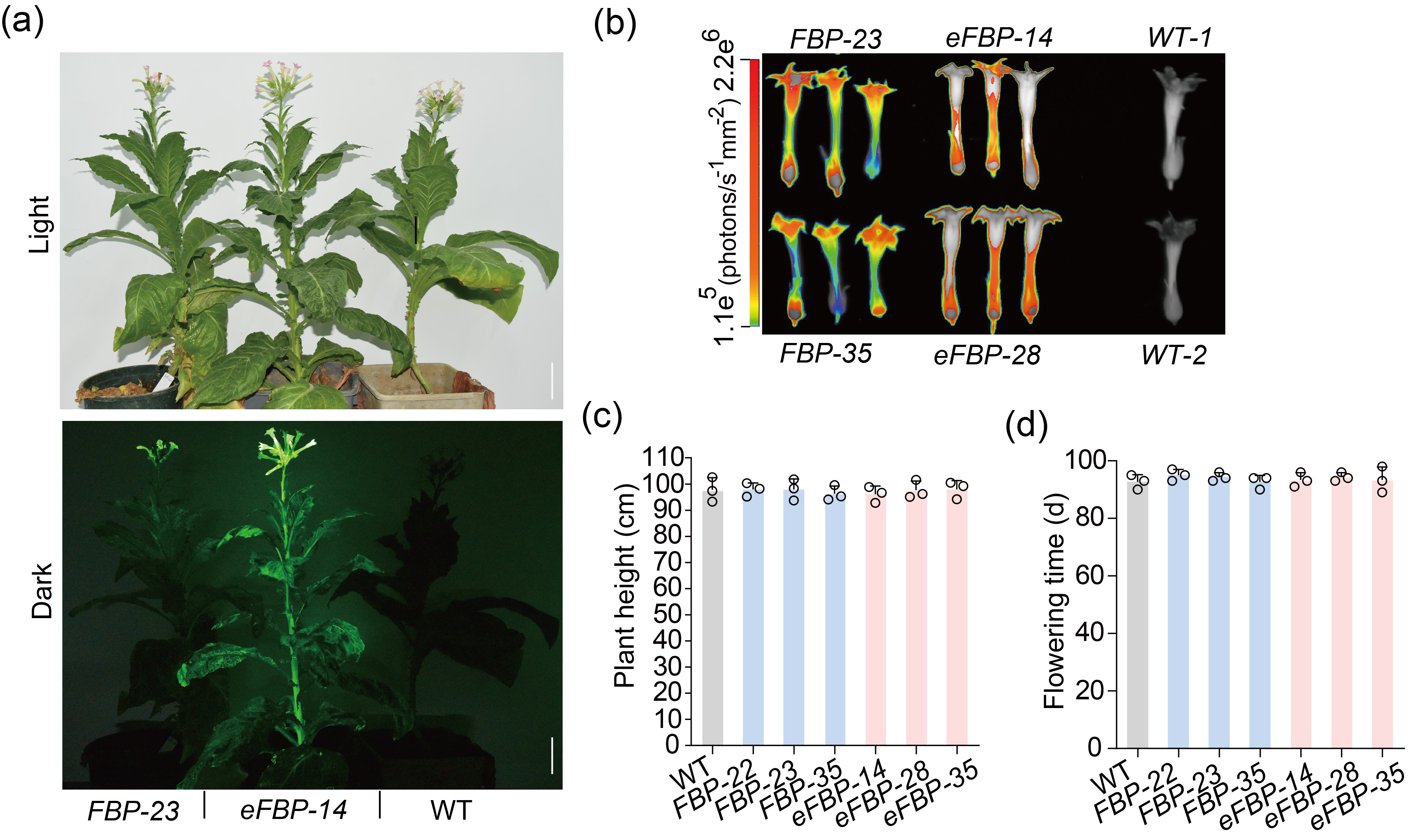

Supplement: Supplementary file 1 — Figure S1 Protein sequences cluster of C3′H homologues. Figure S2 Transiently expressing C3′H1 constructs for enzyme activity assay. Figure S3 Molecular modelling of BnC3′H1. Figure S4 Multiple sequence alignment of C3′H homologues. Figure S5 Identification of the FBP and eFBP DNA modules and transgenic tobacco lines. Figure S6 Identification of the FBP and eFBP transgenic tobacco lines. Figure S7 FBP and eFBP transgenic lines at the flowering stage. Figure S8 Characterization of selectable marker excised plants from eFBP transgenic lines. Figure S9 Analysis of the light emission from FBP and eFBP BY‐2 cell lines. Figure S10 The test of eFBP module to generate luminescence in diverse plant species by transient expression. Figure S11 Identification of eFBP transgenic poplar lines. Figure S12 Analysis of the stability of eFBP transgenic tobacco to abiotic stresses. Figure S13 Oxygen requirement for bioluminescent in eFBP transgenic BY‐2 cells. Figure S14 The stability of photon emission from detached leaves of eFBP transgenic tobacco seedlings. Video S1 The video shows immediate visualization of the auto‐illumination plants in dark room. Table S1 The molecular dockings of p‐Coumaroyl shikimate into the predicted structure of C3'Hs. Table S2 Vectors used in this study. Table S3 Primers used in this study. [file PBI-21-1671-s001.zip › Figure S7.jpg]

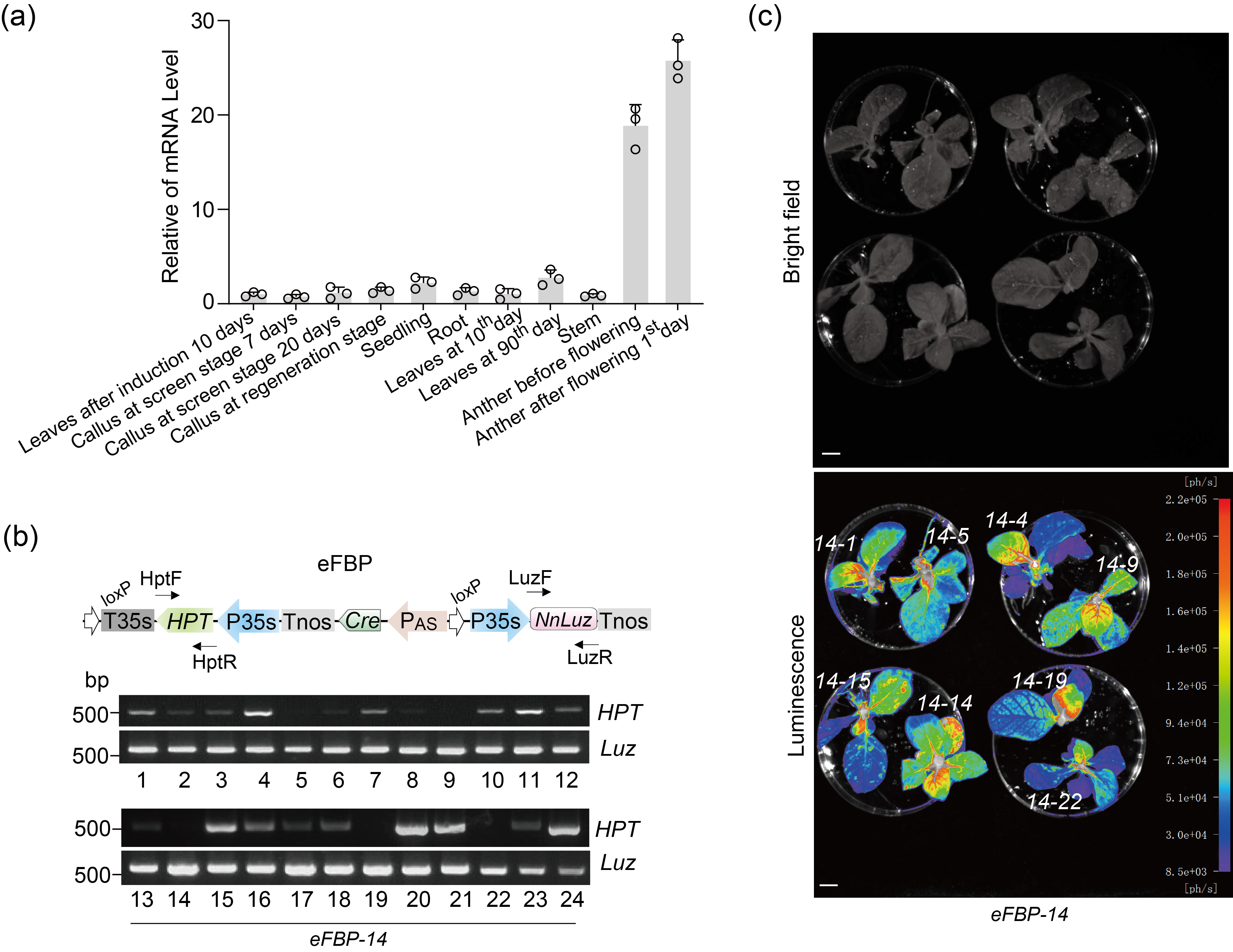

Supplement: Supplementary file 1 — Figure S1 Protein sequences cluster of C3′H homologues. Figure S2 Transiently expressing C3′H1 constructs for enzyme activity assay. Figure S3 Molecular modelling of BnC3′H1. Figure S4 Multiple sequence alignment of C3′H homologues. Figure S5 Identification of the FBP and eFBP DNA modules and transgenic tobacco lines. Figure S6 Identification of the FBP and eFBP transgenic tobacco lines. Figure S7 FBP and eFBP transgenic lines at the flowering stage. Figure S8 Characterization of selectable marker excised plants from eFBP transgenic lines. Figure S9 Analysis of the light emission from FBP and eFBP BY‐2 cell lines. Figure S10 The test of eFBP module to generate luminescence in diverse plant species by transient expression. Figure S11 Identification of eFBP transgenic poplar lines. Figure S12 Analysis of the stability of eFBP transgenic tobacco to abiotic stresses. Figure S13 Oxygen requirement for bioluminescent in eFBP transgenic BY‐2 cells. Figure S14 The stability of photon emission from detached leaves of eFBP transgenic tobacco seedlings. Video S1 The video shows immediate visualization of the auto‐illumination plants in dark room. Table S1 The molecular dockings of p‐Coumaroyl shikimate into the predicted structure of C3'Hs. Table S2 Vectors used in this study. Table S3 Primers used in this study. [file PBI-21-1671-s001.zip › Figure S8.jpg]

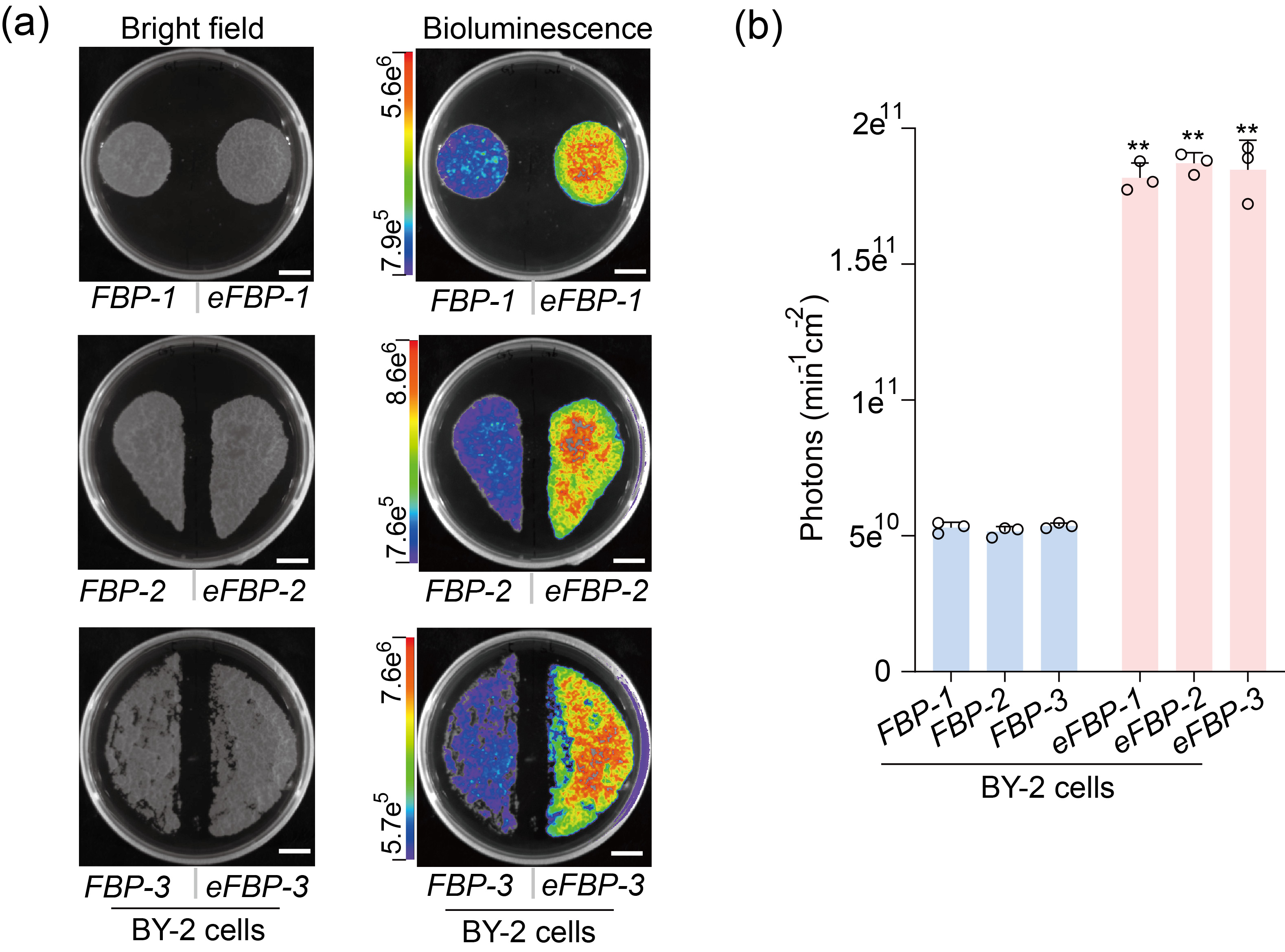

Supplement: Supplementary file 1 — Figure S1 Protein sequences cluster of C3′H homologues. Figure S2 Transiently expressing C3′H1 constructs for enzyme activity assay. Figure S3 Molecular modelling of BnC3′H1. Figure S4 Multiple sequence alignment of C3′H homologues. Figure S5 Identification of the FBP and eFBP DNA modules and transgenic tobacco lines. Figure S6 Identification of the FBP and eFBP transgenic tobacco lines. Figure S7 FBP and eFBP transgenic lines at the flowering stage. Figure S8 Characterization of selectable marker excised plants from eFBP transgenic lines. Figure S9 Analysis of the light emission from FBP and eFBP BY‐2 cell lines. Figure S10 The test of eFBP module to generate luminescence in diverse plant species by transient expression. Figure S11 Identification of eFBP transgenic poplar lines. Figure S12 Analysis of the stability of eFBP transgenic tobacco to abiotic stresses. Figure S13 Oxygen requirement for bioluminescent in eFBP transgenic BY‐2 cells. Figure S14 The stability of photon emission from detached leaves of eFBP transgenic tobacco seedlings. Video S1 The video shows immediate visualization of the auto‐illumination plants in dark room. Table S1 The molecular dockings of p‐Coumaroyl shikimate into the predicted structure of C3'Hs. Table S2 Vectors used in this study. Table S3 Primers used in this study. [file PBI-21-1671-s001.zip › Figure S9.jpg]
